# The Welander TIA1 mutation dedifferentiates insulin-producing cells: Reversal by a GLP-1 receptor agonist

**DOI:** 10.1016/j.jbc.2026.111336

**Published:** 2026-03-03

**Authors:** Tongjian Zhao, Jing Cen, Xuan Wang, Mingyu Yang, Joey Lau, Anders Tengholm, Åke Sjöholm, Nils Welsh

**Affiliations:** 1 Department of Medical Cell Biology, Uppsala University, Uppsala, Sweden; 2 Division of Endocrinology and Diabetology, Department of Internal Medicine, Gävle Hospital, Region Gävleborg, University of Gävle, Gävle, Sweden

**Keywords:** beta cell dedifferentiation, liraglutide, glucagon-like peptide-1, RNA-binding proteins, T-cell intracellular antigen 1, Welander distal myopathy

## Abstract

The RNA-binding proteins TIAR and TIA1 have been reported to affect beta-cell insulin production and viability. The missense E384K TIA1 autosomal dominant mutation is known to cause Welander distal myopathy. This study aimed to study the effects of the TIA1 E384K mutation in human insulin-producing EndoC-βH1 cells. The prime editing technique was used to generate EndoC-βH1 cell clones with the homozygous E384K TIA1 mutation. The E384K TIA1 mutation did not affect high glucose + palmitate-induced stress granule formation and cell death. Instead, the mutated cells respired and proliferated faster than wild-type cells. This was paralleled by a higher *MYC* mRNA and protein level, a profoundly reduced GLP-1 receptor mRNA expression, increased expression of “disallowed” beta cell genes, a proinsulin-to-insulin processing defect, a decreased insulin content and release, a decreased *PAX4/ARX* mRNA ratio, and an increased glucagon production. The TIA1 mutation reduced *MYC* mRNA binding to TIA1. Downregulation of *MYC* mRNA levels normalized insulin/glucagon and *PAX4/ARX* mRNA ratios. Long-term treatment of TIA1-mutated cells with the GLP-1R agonist liraglutide restored insulin production and reversed beta cell dedifferentiation. It is concluded that the TIA1 E384K mutation, *via* increased *MYC* levels and cell proliferation rates, causes beta cell dedifferentiation. Thus, dysfunction of RNA-binding proteins may, at least in certain cases, contribute to the impaired insulin production observed in diabetes. A better understanding of RNA-binding protein-mediated control of beta cell differentiation, and the protective impact of GLP-1 receptor agonism, could facilitate the development of new treatment strategies in diabetes.

In all kinds of diabetes, there is a relative or absolute lack of insulin (1). Insulin production is to a large extent controlled by transcriptional, post-transcriptional, and translational events (2). Disturbances of these events can result in lowered insulin mRNA levels and decreased insulin biosynthesis rates, which may contribute to the development of glucose intolerance and diabetes (2). One cellular process that modulates insulin production rates is the altered binding of RNA-binding proteins (RBP) to insulin mRNA untranslated regions, forming protein complexes that differ in composition and activity (3, 4). The RBPs T-cell intracellular antigen 1 (TIA1) and its paralog T-cell intracellular antigen 1-related (TIAR) are multifunctional proteins involved in many aspects of gene expression control (5, 6). Indeed, TIA1 and TIAR, by binding DNA/RNA sequences, affect mRNA transcription, splicing, localization, translation, and stability (7). TIA1 and TIAR are highly homologous in their amino acid sequence and structure; they both contain three RNA-recognition motifs and six conserved ribonucleoprotein-forming consensus peptide sequences, and they are therefore considered to have similar functions and promote overlapping and redundant effects (5, 6). As both TIA1 and TIAR also contain a prion-like carboxy-terminal domain, which facilitates protein-protein interactions, liquid-liquid phase separation, stress granule formation and pathological fibrillization, these RBPs may play important roles in various neurodegenerative diseases (5, 6). In insulin-producing beta cells, TIAR has been observed to bind untranslated insulin mRNA sequences, leading to decreased insulin mRNA levels and lowered insulin production (7, 8). Furthermore, TIAR was found to accumulate in cytoplasmic stress granules in response to thapsigargin treatment (8), and downregulation of TIA1 expression in INS-1 cells prevented fatty acid-induced stress granule formation (9).

The E384K TIA1 gain-of-function mutation causes Welander distal myopathy, a rare autosomal dominant disease that affects middle-aged patients originating from certain regions in Sweden and Finland (10, 11). The E384K missense mutation is located in the prion-like carboxy-terminal domain, and it has been observed that the mutation affects splicing of survival motor neuron 2 pre-mRNA, stress granule formation dynamics, mitochondrial dynamics, and apoptosis in myoblasts (12, 13). It has also been reported that the E384K TIA1 mutation reduced self-assembly and fibrillization properties, arguing against a role in exaggerated stress granule formation and amyloid deposits (14). Nevertheless, as wild-type TIA1/TIAR seems to affect insulin production and the beta cell response to stress (8, 9), and as the E384K TIA1 mutation promotes pathology in muscle tissue (11), the aim of this study was to introduce the E384K TIA1 mutation into the genome of human insulin-producing EndoC-βH1 cells to better understand how aberrant TIA1 gain-of-function affects beta cell insulin production and cell survival. To this end, we used the prime editing (PE) technique so that any adverse effects of the mutation could be analyzed under conditions with normal endogenous TIA1 gene expression levels, thereby obiviating the inherent problems associated with forced overexpression strategies.

## Results

### Transient overexpression of wild-type (WT) and mutated (Mut) TIA1

WT and Mut (E384K) TIA1, both fused with the GFP-coding sequence and driven by the CMV promoter, were transiently overexpressed in EndoC-βH1 cells. One day after lipofection, the WT TIA1 GFP signal was present in both nuclei and cytoplasm, and showed a granular staining pattern (Fig. S1*A*). Both WT and Mut TIA1 localization partially overlapped with the ER, mitotracker and lysosome probe stains, but the granularity of TIA1 did not co-localize with ER, mitotracker, and lysosome signals. (Fig. S1, *A* and *B*). Thus, both WT and Mut TIA1 seem to be subcellularly compartmentalized to granular structures without specific colocalization with ER, mitochondria or lysosomes.

The TIA1 protein contains a prion-like domain (15). Therefore, we next analyzed whether WT and Mut TIA1 overexpression affected amyloid aggregate formation in EndoC-βH1 cells. Over-night Mut TIA1 overexpression resulted in increased numbers of Congo red aggregates per cell, but again without any clear colocalization between TIA1 immunoreactivity and Congo red (Fig. S1, *C* and *D*). Overexpression of WT TIA1 did not reach statistical significance (Fig. S1*D*).

As Mut TIA1, and possibly also WT TIA1, increased lysosomal and amyloid signals, it is possible that forced overexpression of TIA1 is toxic to EndoC-βH1 cells. We therefore analyzed apoptosis rates 1 day after transfection and observed that it was increased in both WT and Mut TIA1-overexpressing cells (Fig. S1*E*). This occurred despite using a low DNA amount in the lipofection procedure (25 ng) and a short post-lipofection period (18 h).

### TIA1 prime edited EndoC-βH1 cells display unaltered stress granule formation

**Figure 1:**
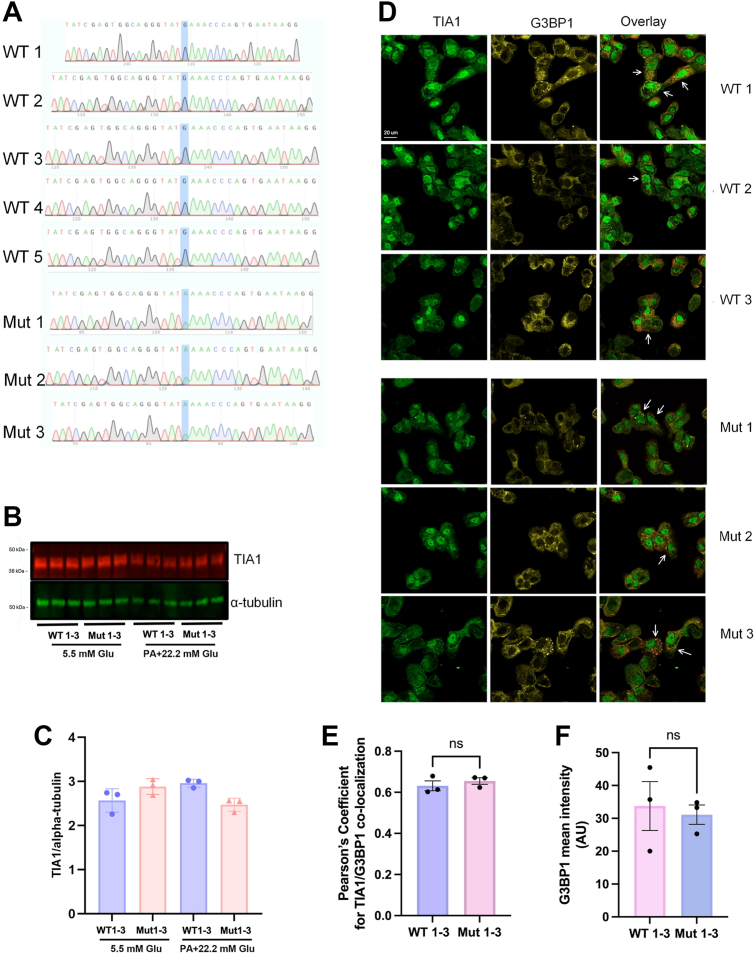
**Verification of E483K TIA1 prime editing in EndoC-βH1 cells, and lack of effects of the TIA1 E483 K mutation on cell stress granules**. *A*, sequencing of wildtype (WT 1–5) and mutant (Mut 1–3) clones was performed after prime editing and clonal selection. The panel depicts sequencing results of the site of the E483 K mutation in the WT and Mut clones. *B*, TIA1 protein of WT 1 to 3 and Mut 1 to 3 EndoC-βH1 cells at control or palmitate + high glucose conditions (18h) were determined by Western blot and quantified in (*C*). One-way ANOVA analysis showed no significant differences between the groups. *D*, TIA1 colocalization with the stress granule marker G3BP1 at palmitic acid + high glucose conditions (1.5 mM PA + 22 mM glucose for 18h) was determined in WT 1 to 3 and Mut 1 to 3 cells by confocal microscopy, and arrows point to G3BP1 positive stress granules that are also TIA1-positive. *E*, cells in (*D*) were analyzed using the ImageJ software for Pearson’s coefficient calculations (TIA1 and G3BP1 intensity correlation from the entire images). Student’s unpaired *t* test was used for statistical comparison. *F*, cells in *D* were analyzed using ImageJ for mean cellular G3BP1 intensity. Student’s unpaired *t* test was used for statistical comparison.

As overexpression of TIA1 was not well tolerated by EndoC-βH1 cells, we instead performed prime editing (PE) to alter the sequence of the chromosomal TIA1 gene. With this approach, overexpression is avoided, and the TIA1 Mut cells express only mutated TIA1, and not a mixture of WT and Mut TIA1. Individual clones were generated, and out of 72 clones that were sequenced, three clones had the TIA1 mutation (Mut 1–3) (Fig. 1*A*). Five WT clones (WT 1–5) from the same gene-editing experiment were included as controls. To establish whether PE affected TIA1 protein levels and size in WT and Mut clones, we performed immunoblot analysis of TIA1. TIA1 migrated similarly in WT and Mut clones, and the protein levels were not different (Fig. 1, *B* and *C*). Furthermore, immunofluorescence staining of TIA1 and G3BP1, a stress granule marker, revealed similar localization of WT and Mut TIA1 at basal conditions (Fig. S2). Both WT and Mut TIA1 were mainly present in nuclei, but showed also a weaker and diffuse pan-cytoplasmic staining pattern. G3BP1, in both WT and Mut cells, was localized to the cytosol with some perinuclear accumulation. As the cells were kept at basal conditions, there were no stress granule structures, and therefore no specific co-localization between TIA1 and G3BP1. We also studied granular colocalization of TIA1 and G3BP1 in WT and Mut clonal cells exposed to palmitate + high glucose for 18 h (Fig. 1*D*), a glucolipotoxic condition that promotes stress granule formation *in vitro* (8). In this case, cytosolic granular structures positive for both TIA1 and G3BP1 were observed in both WT and Mut cells (Fig. 1*D*, white arrows). TIA1 and G3BP1 colocalization was evidenced by a Pearson’s correlation coefficient of approximately 0.6 (Fig. 1*E*), which is a strong correlation considering that TIA1 is present in nuclei, whereas G3BP1 is not. The correlation coefficient was similar in WT and Mut cells. The mean cellular G3BP1 fluorescence intensity was similar in the WT 1 to 3 cells as compared to the Mut 1 to 3 cells (Fig. 1*F*), suggesting that the mutation affected neither accumulation of stress granules in response to palmitate + high glucose nor TIA1 association with stress granules.

### Effects of the TIA1 E483K mutation on proliferation, cell death and mitochondrial function

**Figure 2:**
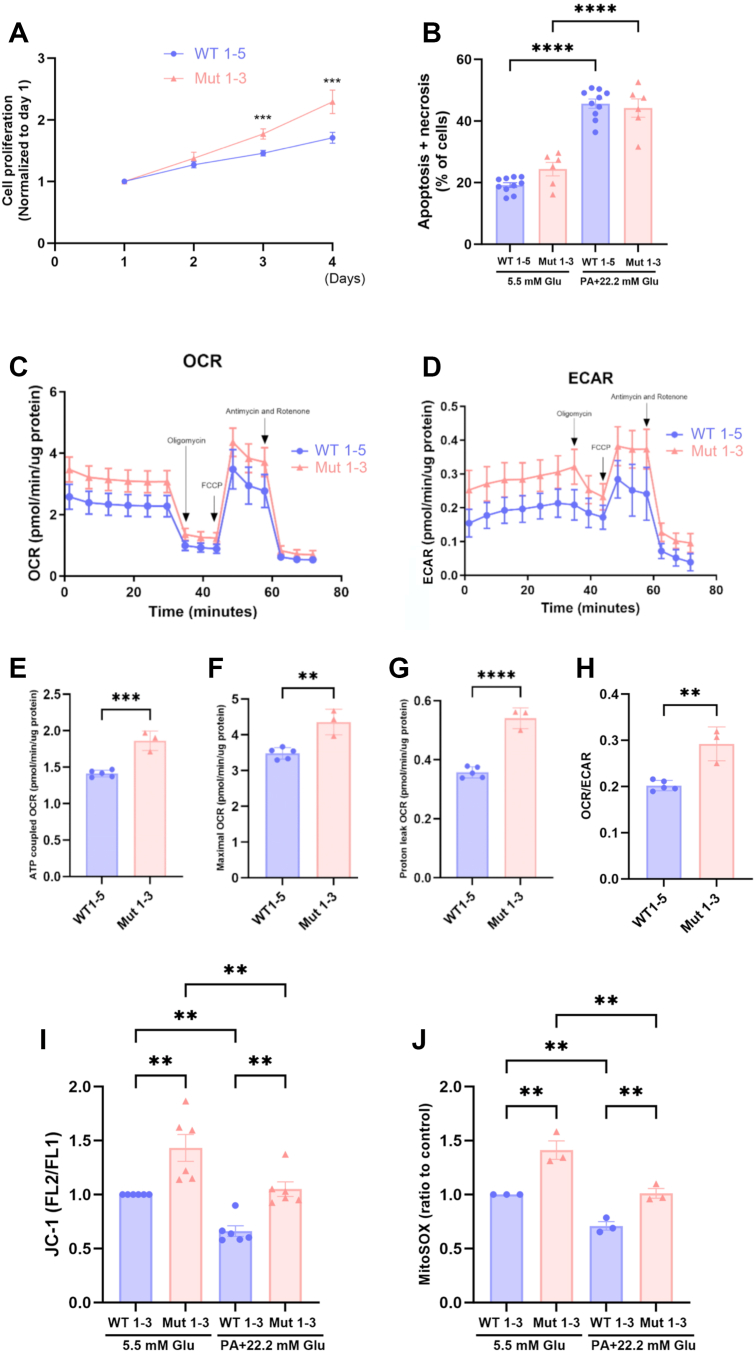
**Effects of the TIA1 E483K mutation on EndoC-βH1 cell proliferation, cell death and mitochondrial function**. *A*, WT 1 to 5 and Mut 1 to 3 clonal cells were plated on day 0 and counted by flow cytometry on days 1 to 4. Results are expressed as ratios to day 1 and are means ± SEM for three independent experiments. ∗∗∗ denotes *p* < 0.001 using a paired Student’s *t* test. *B*, WT 1 to 5 and Mut 1 to 3 clonal cells were cultured for 24 h with or without sodium palmitate (1.5 mM) + 22 mM glucose (P + HG). Cells were then trypsinized and labeled with FITC-Annexin V and propidium iodide, followed by flow cytometry analysis. Results are percentages of Annexin V and/or propidium iodide-positive cells and are expressed as means ± SEM. All clones were analyzed in two independent experiments. ∗∗∗∗ denotes *p* < 0.0001 using one-way ANOVA followed by the Šídák *post hoc* test. *C–H*, WT 1 to 5 and Mut 1 to 3 clonal cells were pre-cultured for 3 days and then analyzed for oxygen consumption rates (OCR) and extracellular acidification rates (ECAR) at 5.5 mM glucose using the Extracellular Flux Analyzer XFe96. Each clone was analyzed using 4 to 6 replicates in one experiment. Results are means ± SEM. ∗∗, ∗∗∗ and ∗∗∗∗ denote *p* < 0.01, 0.001 and 0.0001, respectively, using one-way ANOVA and the Šídák *post hoc* test. *I*, WT 1 to 3 and Mut 1 to 3 clonal cells were pre-cultured for 24 h with or without sodium palmitate (1.5 mM) + 22 mM glucose (P + HG) and then labeled with 4 μΜ JC-1 for 20 min followed by flow cytometric analysis. Clones were analyzed in three independent experiments and results are means ± SEM. ∗∗ denotes *p* < 0.05 using one-way ANOVA and the Šídák *post hoc* test. *J*, WT 1 to 3 and Mut 1 to 3 clonal cells were treated as in panel I followed by labeling with 5 μΜ of the mitoSOX probe. FL2 fluorescence intensity was determined by flow cytometry in four independent experiments. ∗∗ denotes *p* < 0.05 using one-way ANOVA and the Šídák *post hoc* test.

We next characterized phenotypical traits of WT 1 to 5 and Mut 1 to 3 clones. All TIA1 Mut clones exhibited higher proliferation rates than the WT clones (Fig. 2*A*). Cell death rates (apoptosis and necrosis), however, were not different between Mut and WT cells, neither at basal nor at glucolipotoxic conditions (13) (Fig. 2*B*). Typical traces of oxygen consumption rates (OCR) and extracellular acidification rates (ECAR), the latter indicating glycolysis rates, demonstrating responses of WT and Mut cells to oligomycin, FCCP, antimycin + rotenone are shown in Figure 2, *C*–*H*. In these experiments, we observed that OCR, both ATP-coupled and maximal, were higher in Mut cells when compared to WT cells (Fig. 2, *E* and *F*). Proton leak and ECAR were also augmented in Mut 1 to 3 clones (Fig. 2, *G* and *H*). In line with this, mitochondrial inner membrane potential and mitochondrial ROS production were increased in Mut 1 to 3 clones, both at basal conditions and in the presence of palmitate + high glucose (Fig. 2, *I* and *J*).

### Effects of the TIA1 E483K mutation on insulin and proinsulin release and contents

**Figure 3:**
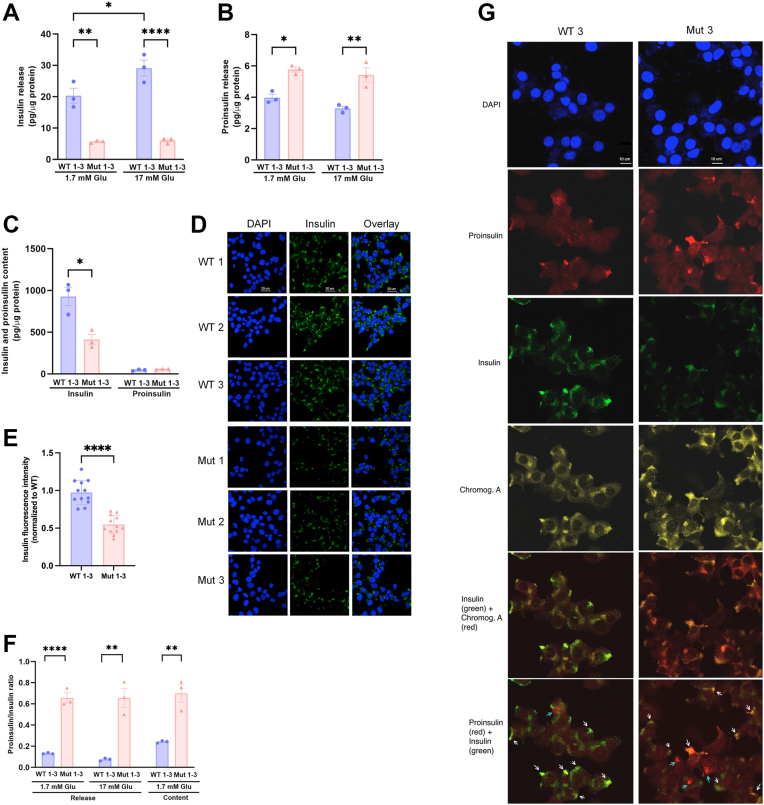
**Effects of the TIA1 E483K mutation on EndoC-βH1 cell insulin and proinsulin release and contents**. *A*, release of insulin during a 30 min incubation period from WT 1 to 3 and Mut 1 to 3 clonal cells at 1.7 and 17 mM glucose was assessed by ELISA. *B*, release of proinsulin during a 30 min incubation period from WT 1 to 3 and Mut 1 to 3 clonal cells at 1.7 and 17 mM glucose was assessed using a proinsulin ELISA. *C*, Insulin and proinsulin contents in cell homogenates were analyzed as in *A* and *B*. *D*, insulin immunofluorescence in WT and Mut clonal cells. WT 1 to 3 and Mut 1 to 3 clonal cells were stained for insulin and analyzed by confocal microscopy. *E*, insulin (green) fluorescence was quantified using ImageJ and expressed as a ratio to wild type clones. *F*, ratios of proinsulin to insulin were calculated from the results in Panels *A*–*C*. Results in panels *A*–*D* are from three independent experiments in which all six clones were analyzed separately. ∗, ∗∗ and ∗∗∗∗ denote *p* < 0.05, 0.01 and 0.0001, respectively, using one-way ANOVA and the Šídák *post hoc* test. *G*, WT 1 to 3 and Mut 1 to 3 clones were co-stained for insulin, proinsulin and chromogranin A and analyzed by confocal imaging. White arrows point to areas in which insulin, proinsulin and chromogranin A co-stain. Blue arrows indicate areas where there is only proinsulin and chromogranin A co-staining. The picture shows representative images from the WT three and Mut three clones.

Mut 1 to 3 cells released less insulin and more proinsulin, at both 1.7 and 17 mM glucose, than WT 1 to 3 cells (Fig. 3, *A* and *B*). While WT cells responded to high glucose with an increased release of insulin, Mut cells did not (Fig. 3*A*). A high glucose concentration failed to increase the release of proinsulin from both WT and Mut cells (Fig. 3*B*). The insulin content, but not the proinsulin content, was lower in Mut clonal cells than in WT cells (Fig. 3*C*). Immunofluorescence staining also revealed a lower insulin content in Mut cells (Fig. 3, *D* and *E*). The lower insulin content and release in Mut cells resulted in an increased proinsulin–to–insulin ratio during secretion at low and high glucose, and in the cellular contents (Fig. 3*F*). These findings suggest that the Welander TIA1 mutation causes a state of impaired beta cell function with lower insulin contents and a glucose-insensitive insulin release, and instead increased prohormone levels.

We next co-immunostained WT 1 to 3 and Mut 1 to 3 cells for insulin, proinsulin and chromogranin A. The secretory vesicle marker chromogranin A colocalized with both insulin and proinsulin in both WT and Mut cells (Fig. 3*G*). This suggests that proinsulin is sorted into secretory vesicles similarly in WT and Mut cells. Colocalization between insulin and proinsulin was strong in WT cells (white arrows), and only a few proinsulin-positive/insulin-negative areas could be observed (blue arrows). In Mut cells, however, more proinsulin-positive/insulin-negative areas were observed. By counting the number of proinsulin-positive/insulin-negative areas per total number of insulin- or proinsulin-positive areas, we observed that the relative frequency of proinsulin-positive/insulin-negative areas was 0.10 ± 0.04 in WT cells and 0.53 ± 0.03 in Mut cells (*p* < 0.0001 using Student’s *t* test; 20–30 cells from each clone analyzed). This aligns well with the increased proinsulin-to-insulin content ratios observed by ELISA analysis in Mut cells (Fig. 3*F*).

### Effects of the TIA1 E483K mutation on the EndoC-βH1 transcriptome and handling of Ca^2+^ and cAMP

**Figure 4:**
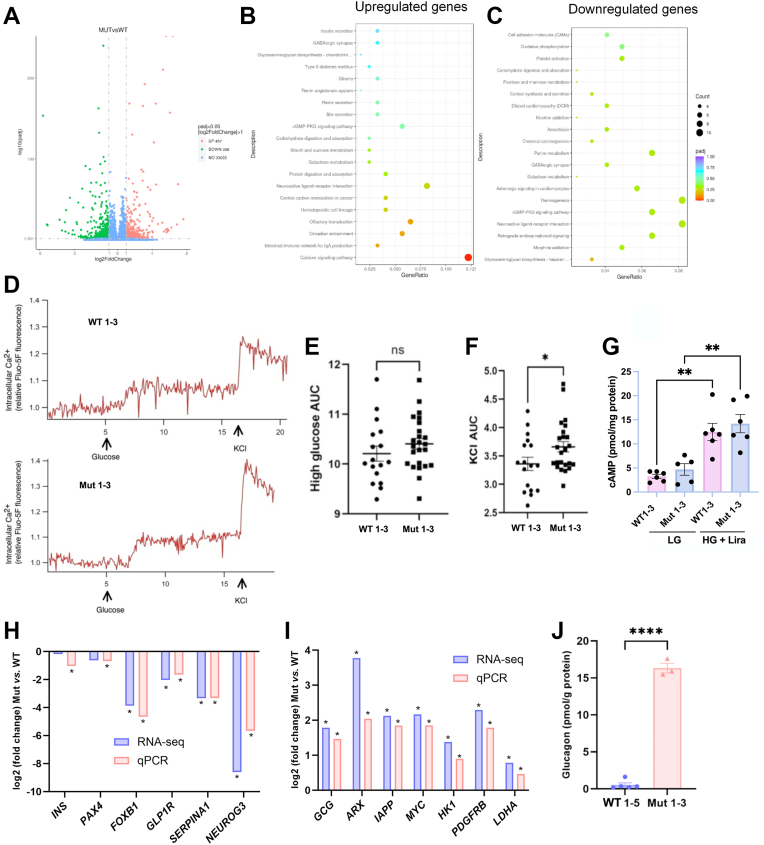
**Differential gene expression caused by the E483K TIA1 mutation—lack of effect on glucose-induced Ca^2+^ and cAMP responses**. *A*, Volcano map of all differentially expressed genes in the Mut 1 to 3 and WT 1 to 5 clones, as assessed by RNAi. *B*, Upregulated and (*C*) downregulated genes were analyzed using the KEGG (Kyoto Encyclopedia of Genes and Genomes) pathway analysis tool. *D*, sub-membrane Ca^2+^ recordings from WT 1 to 3 and Mut 1 to 3 cells stimulated by an increase of the glucose concentration from 1.7 to 17 mM and membrane depolarization with 100 mM KCl. Traces are averaged from single-cell recordings from 17 WT and 24 Mut cells. *E* and *F*, glucose- and KCl-induced Ca^2+^ responses quantified as the area under the curve (6.2–15.8 min for glucose; 16.2–19 min for KCl). ∗ denotes *p* < 0.05 using Student’s *t* test. *G*, cAMP levels, assessed by ELISA, in WT 1 to 3 (WT) and Mut 1 to 3 (Mut) cells, exposed to 1.7 mM or 17 mM glucose + 100 nM liraglutide for 30 min ∗∗ denotes *p* < 0.01 using one-way ANOVA and the Šídák *post hoc* test. The downregulated (*H*) and upregulated (*I*) genes, as assessed by RNA-seq and qPCR verification, are shown and results are expressed as log2 fold change. For absolute values ± SEM and detailed statistical analysis, please see Table S2. ∗ denotes *p* < 0.05 using the FDR test (RNA-seq) or Student’s *t* test (qPCR). All clones were analyzed on two separate occasions. *J*, the glucagon contents in WT and Mut cells were analyzed by ELISA. Each clone was analyzed using 4 to 6 replicates in three experiments. Results are means ± SEM. ∗∗∗∗ denote *p*< 0.0001, respectively, using one-way ANOVA and the Šídák *post hoc* test.

The five WT 1 to 5 and the three Mut 1 to 3 clones, each in duplicates, were RNA-sequenced after culture at basal conditions. Raw data sequencing results are uploaded onto ArrayExpress (accession number E-MTAB-16316). Differential gene expression analysis revealed that 398 genes were upregulated by more than 100% and 457 genes were downregulated by more than 50% using an FDR *p*-value of <0.05 (Table S1, Fig. 4*A*). KEGG pathway analysis of upregulated genes revealed significant clustering of differentially expressed genes belonging to the Ca^2+^ signaling pathway (15 genes) (Fig. 4*B*). The *p*-values of the remaining pathways/categories were above 0.05 and therefore of low significance. Also, the KEGG pathways/categories obtained with the downregulated genes were of low significance (Fig. 4*C*). As the KEGG analysis suggested altered Ca^2+^ signaling, we investigated sub-plasma membrane signaling relevant for insulin secretion in WT 1 to 3 and Mut 1 to 3 clonal cells. We observed a small glucose (17 mM)-induced increase in cytosolic Ca^2+^ in both WT 1 to 3 and Mut 1 to 3 cells (Fig. 4*D*), but the glucose response measured as area under the curve was not affected by the TIA1 mutation (Fig. 4*E*). Both WT 1 to 3 and Mut 1 to 3 cells responded to depolarization with KCl (100 mM) with a prompt increase in Ca^2+^ that was slightly more pronounced in Mut cells (Fig. 4*F*). We also analyzed cAMP levels and observed no differences in cAMP when comparing WT and Mut cells, neither at 1.7 mM glucose nor at 17 mM glucose + 100 nM liraglutide (Fig. 4*G*). Together, these results demonstrate that Ca^2+^ and cAMP signaling is largely preserved in cells with the TIA1 mutation.

Next, genes important for beta cell function were selected for validation with qPCR (Fig. 4, *H* and *I*, Table S2). Transcripts for *INS*, *PAX4*, *FOXB1*, *GLP1R*, *NEUROG3* and *SERPINA1* were downregulated in Mut 1 to 3 cells (Fig. 4*H*), whereas the transcripts for *GCG*, *ARX*, *IAPP* and *MYC* were upregulated (Fig. 4*I*). Also of interest is that the “disallowed” beta cell genes *HK1*, *LDHA1* and *PDGFRB* were all upregulated in Mut clonal cells (Fig. 4*I*, Table S2). These findings suggest that the *TIA1* mutation promotes beta-cell dedifferentiation and partial beta-to-alpha cell transdifferentiation. To verify this beta-to-alpha transdifferentiation, we analyzed glucagon contents in WT and Mut cells. No glucagon could be detected in clones WT 1 to 5. In clones Mut 1 to 3, however, glucagon levels were 16.3 ± 0.7 pmol/g protein (*p* < 0.0001 *versus* WT, Student’s *t* test) (Fig. 4*J*), which corresponds to 57.1 ± 2.3 ng glucagon/g protein. When comparing the molar ratio between insulin and glucagon in Mut 1 to 3 cells, insulin contents are approximately 3000 times higher than corresponding glucagon contents. We also attempted to quantify glucagon release to the incubation medium, but levels were below the detection limit of the ELISA (not shown).

Transcriptome analysis also revealed that the TIA1 mutation promoted alternative splicing of 79 genes (Table S3). SNP analysis identified numerous mutations in the different WT and Mut clones, due to clonal variation, but only the TIA1 mutation was consistently altered in Mut clones when compared to WT clones (results not shown).

### The TIA1 mutation increases MYC protein levels, reduces TIA1 binding to *MYC* mRNA, and participates in EndoC-βH1 cell dedifferentation

**Figure 5:**
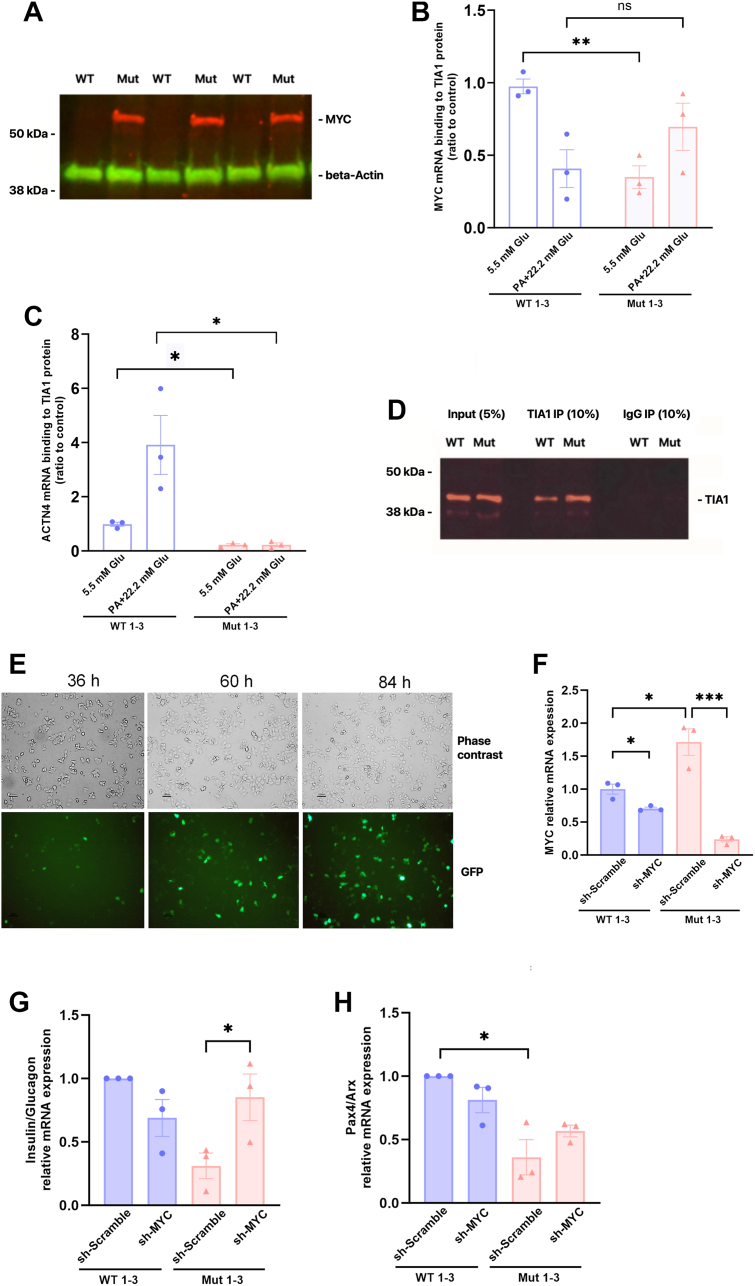
**The TIA1 E384K mutation impairs *MYC* mRNA binding, while anti-*MYC* shRNA restores β-cell identity**. *A*, WT 1 to 3 (WT, a mixture of WT clones 1–3) and Mut 1 to 3 (Mut, a mixture of Mut clones 1–3) cell levels of MYC protein were visualized by immunoblot analysis. Beta-actin was used as a loading control. The Figure shows harvested at three different occasions. *B*–*D*, WT 1 to 3 (WT, a mixture of WT clones 1–3) and Mut 1 to 3 (Mut, a mixture of Mut clones 1–3) cells, with and without treatment with palmitate (1.5 mM) and high glucose (22 mM), were RNA-immunoprecipitated using an anti-TIA1 antibody. RNA molecules binding to the TIA1 protein were isolated and quantified using RT-PCR. (*B*) *MYC* mRNA binding to TIA1. *C*, ACTN4 mRNA binding to TIA1. *D*, immunoblot showing immunoprecipitation of TIA1. Results are from one experiment with three replicates and are shown as means ± SEM. ∗, and ∗∗ denote *p* < 0.05 and 0.01, respectively, using two-way ANOVA and the Šídák *post hoc* test. *E*, WT 1 to 3 (WT, a mixture of clones 1–3) and Mut 1 to 3 (Mut, a mixture of clones 1–3) clone cells were transduced with lentiviral vectors (scramble and anti-*MYC* shRNA, 25 MOI). Transduction efficacy was visualized by GFP-fluorescence microscopy at 36, 60 and 84 h post transduction. Upper row (phase contrast) shows total number of cells and lower row (GFP) shows transduced cells. *F*, Lentivirally transduced WT 1 to 3 cells (mixture of clones 1–3) and Mut 1 to 3 cells (mixture of clones 1–3) were analyzed in three separate experiments for *MYC*/beta actin mRNA levels 96 h post-transduction. Results were normalized to the WT 1 to 3 scramble group. *G*, cells were analyzed as in F for insulin/glucagon mRNA ratios. *H*, cells were analyzed as in F for *PAX4/ARX* mRNA ratios. ∗, ∗∗∗ and ∗∗∗∗ denote *p* < 0.05, 0.001 and 0.0001, respectively, using one-way ANOVA and the Šídák *post hoc* test.

TIAR is known to target the 3-UTR of *MYC* mRNA resulting in decreased *MYC* expression (16). Having observed that TIA1 Mut cells proliferate faster than WT cells (Fig. 2*E*) and express higher levels of *MYC* mRNA (Fig. 4*I*, Table S2), a transcription factor that drives beta cell proliferation and dedifferentiation (17), we analyzed MYC protein levels in WT 1 to 3 and Mut 1 to 3 cells cultured at basal conditions. Immunoblot analysis revealed that MYC protein could not be detected in WT 1 to 3 cells (Fig. 5*A*). In Mut 1 to 3 cells, however, a clear MYC band, migrating as a 65 kDa protein, was observed. In view of this marked induction of the MYC protein, we hypothesized that the TIA1 mutation abolishes TIA1-induced *MYC* downregulation. A mixture of WT 1 to 3 and Mut 1 to 3 clonal cells were RNA-immunoprecipitated with a TIA1 antibody, both at basal and metabolic stress conditions (palmitate + high glucose), and *MYC* mRNA binding to TIA1 was assessed by RT-PCR. We also assessed alpha-actinin-4 (*ACTN4*) mRNA binding to TIA1, as this is a previously verified TIA1 target in neuronal cells (18). We observed that the E483 K mutation reduced binding of both *MYC* and *ACTN4* mRNA to TIA1 (Fig. 5, *B* and *C*). Metabolic stress seemed to affect TIA1 mRNA binding affinities in WT cells as *ACTN4* mRNA binding appeared stronger (*p* = 0.055, Student’s *t* test) and *MYC* mRNA binding was weaker (*p* = 0.011, Student’s *t* test). In Mut cells, however, *MYC* mRNA and *ACTN4* mRNA binding to TIA1 remained unaffected in the presence of palmitate + high glucose. The immunoprecipitation efficiency is shown in Figure 5*D*. These results suggest that the TIA1 mutation increases *MYC* mRNA levels by alleviating the mRNA from TIA1-induced expression and translational silencing, and that metabolic stress mimics somewhat the effects of the mutation.

To verify that the TIA1 mutation-induced increase in *MYC* expression participated in EndoC-βH1 cell dedifferentiation, we transduced WT and Mut cells with shRNA lentiviruses that either contained scrambled or anti-*MYC* sequences. GFP-tagging of the shRNA constructs revealed increasing numbers of transduced cells, at 36, 60 and 84 h post-transduction, reaching a transduction efficiency of approximately 50% (Fig. 5*E*), which prompted us to harvest cells at 96 h for qPCR analysis. Analysis of *MYC* mRNA levels showed that the anti-*MYC* shRNA construct reduced *MYC* mRNA levels in both WT and Mut clonal cells (Fig. 5*F*). The Mut 1 to 3 clones displayed markedly decreased insulin/glucagon and *PAX4/ARX* ratios (Fig. 5, *G* and *H*). The Mut cells treated with anti-*MYC* shRNA restored their insulin/glucagon mRNA ratios so that they became similar to those of WT clones (Fig. 5*G*). The *PAX4/ARX* mRNA ratio was reduced in Mut cells treated with scrambled shRNA, but not in Mut cells treated with anti-*MYC* shRNA (Fig. 5*H*). These findings suggest that the TIA1 mutation promotes beta cell trans- or dedifferentiation, at least in part, *via* increased *MYC* expression.

### GLP-1 receptor agonist liraglutide reverses TIA1 mutation-induced beta cell dedifferentiation

**Figure 6:**
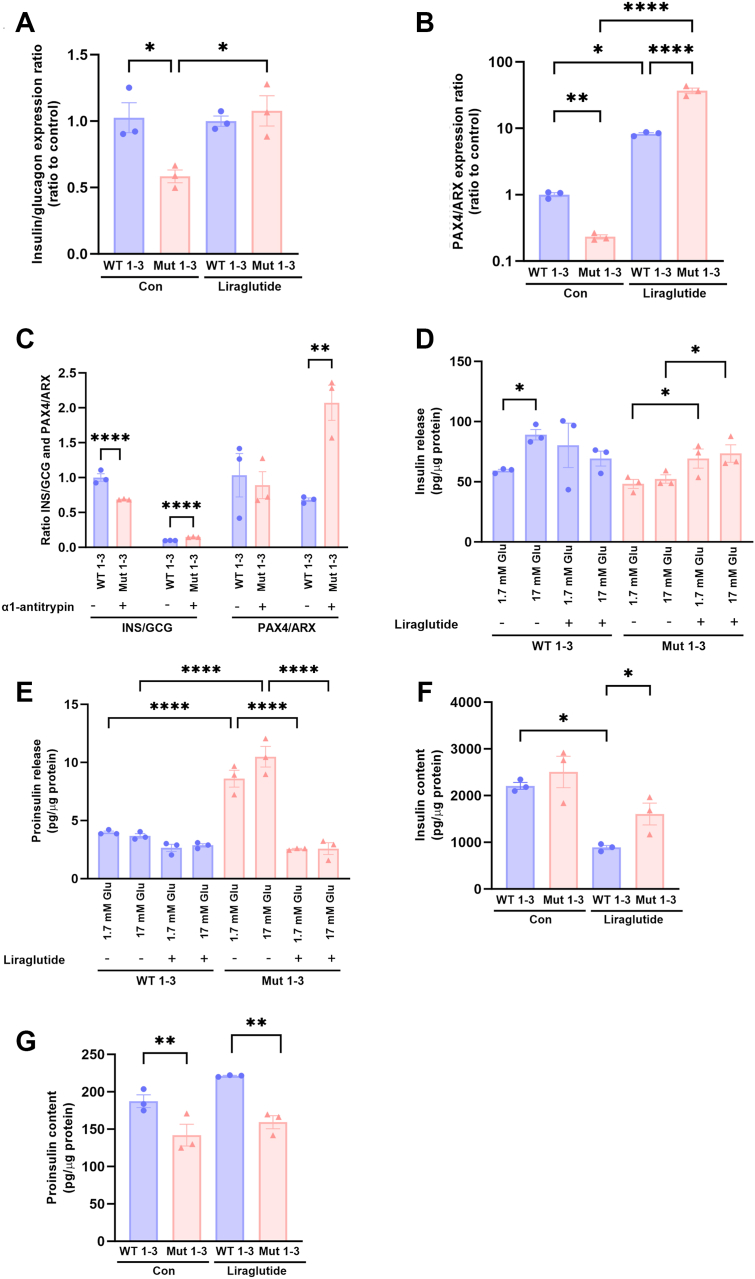
**GLP-1 receptor agonist liraglutide reverses TIA1 mutation-induced transdifferentiation**. WT 1 to 3 clones and Mut 1 to 3 clones were cultured for 2 weeks with or without 100 nM liraglutide and then used for qPCR analysis of insulin, glucagon, *PAX4* and *ARX* mRNA levels. Results are expressed as (*A*) insulin to glucagon mRNA ratios, and (*B*) *PAX4* to *ARX* mRNA ratios, and were normalized to the WT control group. Results in panels *A* and *B* are from three independent experiments. *C*, WT 1 to 3 and Mut 1 to 3 clones were cultured for 4 days with or without 0.5 mg/ml alpha1-antitrypsin and then used for qPCR analysis of insulin, glucagon, *PAX4* and *ARX* mRNA levels. Results are from three independent experiments and shown as means ± SEM. ∗, ∗∗ and ∗∗∗∗ denote *p* < 0.05, 0.01 and 0.0001, respectively, using one-way ANOVA and the Šídák *post hoc* test. *D*, WT 1 to 3 clones and Mut 1 to 3 clones were cultured for 2 weeks with or without 100 nM liraglutide and then used for (*D*) insulin or (*E*) proinsulin release experiments at 1.7 and 17 mM glucose. *F*, Insulin and (*G*) proinsulin contents were quantified in cell homogenates using specific ELISAs. Results are from three independent experiments and shown as means ± SEM. ∗, ∗∗ and ∗∗∗∗ denote *p* < 0.05, 0.01 and 0.0001, respectively, using one-way ANOVA and the Šídák *post hoc* test.

We next determined whether the clinically widely used GLP-1 receptor agonist, liraglutide, counteracts beta cell dedifferentiation as has been reported to occur in diabetes (19). The transcription factor PAX4 is a marker for beta cell differentiation, whereas ARX is a marker for alpha cell differentiation (20). The insulin/glucagon and *PAX4/ARX* mRNA ratios were clearly decreased in Mut 1 to 3 cells (Fig. 6*A*). Liraglutide completely restored insulin/glucagon mRNA ratios (Fig. 6, *A* and *B*). In addition, *PAX4/ARX* mRNA ratios were dramatically increased by liraglutide both in WT 1 to 3 and Mut 1 to 3 cells (Fig. 6*B*).

As the expression of the alpha1-antitrypsin gene *SERPINA1* was reduced in TIA1-mutated cells (Fig. 4*G*, Table S2), we also attempted to restore beta cell differentiation by culture in the presence of alpha1-antitrypsin. Alpha1-antitrypsin is an acute phase anti-inflammatory protease inhibitor that has been observed to counteract beta cell dysfunction in diabetic mice (21). While we observed increases in the insulin/glucagon and *PAX4/ARX* mRNA ratios of Mut 1 to 3 cells (Fig. 6, *B* and *C*), the effects were clearly weaker than those observed using liraglutide (Fig. 6, *A* and *B*).

In T1D, although insulin positivity is lost, remaining islet cells persistently stain for proinsulin, and in some cases, the same islet cells express both proinsulin and glucagon (22). Therefore, as beta cell loss of differentiation in diabetes may involve not only increased *ARX* mRNA and glucagon levels, but also defective proinsulin processing, we next verified the beneficial effects of liraglutide on beta cell differentiation by quantifying insulin and proinsulin release. Liraglutide did not affect insulin release from WT cells (Fig. 6*D*). In Mut cells, insulin release at both 1.7 and 17 mM glucose was increased. However, this did not lead to restoration of glucose-stimulated insulin release. Proinsulin release was markedly reduced by liraglutide in Mut cells (but not in WT cells), at both glucose concentrations (Fig. 6*E*). Liraglutide increased insulin and decreased proinsulin contents in Mut cells (Fig. 6, *F* and *G*). Also in WT cells liraglutide somewhat reduced proinsulin contents (Fig. 6*G*). Overall, liraglutide normalizes not only insulin to glucagon ratios but also insulin and proinsulin secretion so that the TIA1-mutated clonal cells regain much of the secretory phenotype of WT cells. These findings lend support to previous reports showing that GLP-1 receptor agonists may enhance proinsulin processing (23).

## Discussion

We report here successful PE-mediated genomic modification of human EndoC-βH1 cells, which are considered a suitable model for adult primary beta cells (24, 25, 26). By introducing the homozygotic Welander E384 K point mutation to the genome of these cells, we have been able to study an aberrant gain-of-function version of the RBP TIA1, without the inherent problems associated with traditional overexpression strategies. Indeed, our initial approach to overexpress WT and TIA1 mutated constructs resulted in general toxicity and was therefore not a meaningful strategy. Using the PE technique, however, we obtained three E384K TIA1 clones with no other consistent point mutations or genetic rearrangements.

Our finding that the TIA1 E384K mutation did not affect beta cell death, neither at basal conditions nor at metabolic stress conditions, and that the mutation increased respiration and proliferation rates, argues against the notion that mutated TIA1 promotes a general stress granule- and/or fibrillization-induced toxicity. This fits well with the recent observation that the E384 K mutation of TIA1 does not result in increased self-assembly and fibrillization (14). Instead, it is likely that the mutation, which is located in the carboxy-terminal protein interaction domain, affects specific protein-to-protein or protein-to-mRNA interactions resulting in moderate phenotypic alterations. One such critical interaction is probably that with *MYC* mRNA. *MYC* mRNA is expressed at very low levels in WT EndoC-βH1 cells, but in TIA1-mutated cells, it was increased 4-fold. Therefore, as TIAR is known to bind to the 3′-UTR of *MYC* mRNA and suppress its expression (26), and as we presently observed weakened *MYC* mRNA binding to the mutated TIA1 protein, it is probable that the increased *MYC* mRNA levels in mutated cells were caused by amelioration of TIA1-induced *MYC* mRNA silencing. Furthermore, MYC protein levels were more or less undetectable in WT cells but clearly visible in Mut cells, suggesting that wildtype TIA1 suppresses not only MYC mRNA levels but also translation of the MYC protein.

Adult human beta cells are normally either post-mitotic or very slowly proliferating cells (27). Increased proliferation can, however, be achieved in response to forced *MYC*-upregulation (28), but when this occurs in postnatal beta cells, it is often associated with dedifferentiation (29, 30, 31). Indeed, we presently observed numerous signs of beta cell dedifferentiation, such as increased expression of several “disallowed” genes, profoundly decreased GLP-1 receptor expression, a reduced *PAX4/ARX* mRNA ratio, a proinsulin-to-insulin processing defect, increased glucagon production and an impaired insulin release. As anti-*MYC* shRNA lentiviral treatment restored *PAX4/ARX* ratios in the TIA1 Mut cells, it is likely that the *MYC*-driven proliferation, at least in part, promoted the loss of beta cell differentiation.

We have previously reported that TIAR binds to the insulin mRNA 3′-UTR and that this results in reduced insulin mRNA levels and lowered insulin biosynthesis rates (8). It is not clear from the present results whether the E384K TIA1 mutation affected this particular process. Admittedly, we observed a modest decrease in insulin mRNA in TIA1 Mut cells, but this might have occurred secondarily to the dedifferentiation process and not *via* a direct effect on the TIA1-insulin mRNA interaction. Thus, the E384K TIA1 mutation may affect TIA1 interactions with insulin and *MYC* mRNA differently.

It appears that an isolated and strong overexpression of *MYC* promotes not only increased beta cell proliferation and respiration, but also dedifferentiation and apoptosis (17). On the other hand, a more modest induction of *MYC*, coupled with receptor activation of other signaling systems, appears to stimulate proliferation without loss of differentiation (17, 32). It is not clear which co-stimulatory events counteract the negative effects of *MYC* upregulation, but it has been suggested that the Wnt receptor Fzd9 mediates *MYC*-induced beta cell dedifferentiation (33). In such a scenario, signaling events that reduce Fzd9 expression might modulate *MYC*-induced effects so that dedifferentiation does not occur. In the present investigation, however, the TIA1-mutated cells underwent dedifferentiation, suggesting that the overall E384K-induced effects did not counterbalance the effects of the increased *MYC* levels.

Increased glucagon production is a prominent feature in both T1D and T2D (34). Although glucagon was discovered > 100 years ago, it has been very much underappreciated in comparison to insulin despite the fact that its pathogenic role in diabetes was early recognized (35). However, recent years have witnessed a remarkable renewed interest in glucagon as a therapeutic target that could be amenable to pharmacological (36, 37, 38, 39) and genetic (40, 41) manipulation. The present findings support the notion that an altered TIA1/MYC mRNA interaction promotes adult beta cells to undergo partial dedifferentiation into insulin/glucagon double-positive cells.

Enhanced incretin signaling *via* the GLP-1 receptor promotes anti-diabetic effects by, among other things, enhancing beta cell differentiation (19), and the dedifferentiation of the TIA1-mutated cells might therefore have developed as a consequence of a lowered GLP-1 receptor expression and/or signaling. The GLP-1 receptor is known to signal *via* increased cAMP and intracellular Ca^2+^ (42). At basal and high glucose + GLP-1-stimulated conditions, cAMP and intracellular Ca^2+^ levels and responses were not reduced in the TIA1 E384 K cells, suggesting that GLP-1 receptor signaling is functional despite decreased receptor expression. Furthermore, as liraglutide potently restored the *PAX4/ARX* ratio and insulin production in TIA1-mutated cells, it is likely that maximal GLP-1 receptor activation, also in cells with a diminished receptor expression, counteracts *MYC*-induced dedifferentiation. Thus, it may be that lowered GLP-1 receptor expression is not a primary cause of beta cell dedifferentiation, and that increased GLP-1 receptor signaling permits and enhances *MYC*-dependent beta cell proliferation without promoting loss of insulin production (32). Interestingly, stimulation of the GLP-1 receptor has been observed to counteract beta cell dysfunction in another monogenic diabetes condition, the Wolfram Syndrome 1 (43), but in that case, by alleviation of ER stress. Therefore, it may be that GLP-1 receptor stimulation rescues beta cells suffering from genetic defects affecting different cellular processes.

As concluding remarks, we would like to point out that not only *MYC* seems to promote an opposite relationship between proliferation and differentiation, as we have previously observed that the transcription factor Zinc Finger BED-Type Containing 6 (ZBED6) maintains beta cell proliferation at the expense of certain specialized functions (44). It is also noteworthy that the phenotype of the TIA1-mutated cells clearly resembles that of beta cells in early T1D and T2D, making it a possible *in vitro* model of the disease *in vivo*. The finding that a clinically used GLP-1 receptor agonist was found to normalize virtually all aberrations evoked by the Welander TIA1 mutation is also very encouraging from a therapeutic perspective. In a future perspective, it remains to be determined whether the E384 K TIA1 mutation affects *MYC* expression in muscle or neuronal cells of Welander patients, and whether Welander myopathy in patients involves similar mechanisms as those involved in beta cell dedifferentiation. Finally, it is also unclear whether glucose intolerance or diabetes occurs at higher frequencies in Welander patients; however, a clinical study addressing this question is currently underway.

In conclusion, we presently report that the Welander E384 K TIA1 mutation in human insulin-producing EndoC-βH1 cells promotes *MYC*-induced dedifferentiation of these cells into a diabetogenic phenotype, and that this process is counteracted by liraglutide. The similarity of the E384 K mutated cells with insulin-producing cells in early stages of T1D and T2D suggests that RBP dysfunction may contribute to the pathogenesis of diabetes. A better understanding of RBP-mediated control of beta cell differentiation, and the protective impact afforded by GLP-1 receptor agonism, could facilitate the development of new treatment strategies against diabetes.

## Experimental procedures

### EndoC-βH1 cell culture

Human EndoC-βH1 cells, obtained from Human Cell Design, Toulouse, France, were cultured in Geltrex Basement Membrane Matrix (Thermo Fisher Scientific)-coated culture flasks in Ham’s F12/DMEM (50%/50%, vol/vol, 5.5 mM glucose) with additional supplements as previously given (24, 45). Cells were cultured at 37 °C in a humidified incubator containing 5% CO_2_, and were kept at mycoplasma-free conditions. Cells were used at passage numbers 65–80, and no more than 10 passages after clonal selection of the CRISPR/Cas9-edited cells.

### Prime editing (PE) in EndoC-βH1 cells

Single nucleotide replacement in the human *TIA1* gene (E384 K) in human EndoC-βH1 cells was achieved by PE according to a previous publication (46). The PE3 system was chosen because it applies preferential repair of nicked DNA strands to enhance PE efficiencies. CRISPR PE guide RNA (pegRNA) and nicking sgRNA (ngRNA) sequences were designed using the web resources available at https://drugthatgene.pinellolab.partners.org/primevar. The target sequences and related synthesized oligonucleotides for pegRNA and ngRNA are shown in Table S4. Oligos were synthesized by Eurofins Genomics. PE plasmid is pCMV-PE2-P2A-GFP (Addgene plasmid #132776) expressing Cas9 H840 A with co-translational GFP expression. PegRNA plasmid was constructed by ligation of annealed oligonucleotides including sequences of spacer, scaffold, and 3′ extension, into BsaI-digested pU6-pegRNA-GG-acceptor (Addgene plasmid # 132777). Plasmids expressing ngRNAs were constructed by ligation of annealed oligonucleotides into U6-BsmBIcassette-Sp-sgRNA BPK1520 (Addgene plasmid # 65777) (47).

EndoC-βH1 cells were seeded in Geltrex Matrix coated T25 flasks. Cells were transfected at approximately 60% confluency with 5 μl Lipofectamine 2000 (Thermo Fisher Scientific) together with 3.6 μg PE plasmid, 1.2 μg pegRNA plasmid, and 0.4 μg ngRNA plasmid according to the manufacturer’s instructions. Cells were cultured for 3 days followed by sorting of GFP-positive cells (approximately 10%) by flow cytometry. GFP-positive cells were seeded in Geltrex-coated 6-well plates at various cell densities and cultured for another 5 weeks. At intermediate cell densities, individual colonies developed (five wildtype (WT 1–5 and three mutated (Mut 1–3) that were picked and re-seeded into a 96-well plate for 7 days, followed by reseeding into two wells of 96-well plates for each clone. One well of the cells from an individual clone was used for DNA isolation (Qiagen) and PCR amplification of the amplicon covering the TIA1 mutation site. The PCR product was then sequenced at Eurofins Genomics for detection and confirmation of the homozygous point mutation (G > A on the sense strand, E384K).

### Immunoblot analysis

WT 1 to 5 and Mut 1 to 3 clone cells were washed with ice-cold PBS, then lysed in SDS sample buffer containing β-mercaptoethanol, boiled for 5 min and separated by pre-cast 4 to 20% SDS-PAGE gels (Bio-Rad). Proteins were then transferred onto a Hybond-PVDF membrane (GE Healthcare). After blocking 1h with 2% bovine serum albumin (BSA), the filter was incubated with the primary antibodies: rabbit anti-TIA1 (1:1000, Cell Signaling Technology), mouse anti-alpha tubulin (1:1000, Santa Cruz), mouse anti-beta-actin (1:1000, Santa Cruz), rabbit anti-c-Myc (1:1000, Cell Signaling Technology). Fluorescent anti-mouse/rabbit/rat secondary antibodies were used (1:15,000, LI-COR Biosciences). All antibodies used were verified by the commercial suppliers. The filters were visualized and quantified with an LI-COR Odyssey Fc system (LI-Cor Biosciences). Band densities were normalized to α-tubulin or β-actin. Data were shown as relative fold change compared with WT cells.

### Cell proliferation

5 × 10^3^ cells from 5 EndoC-βH1-WT (WT 1–5) and 3 EndoC-βH1-mutant (Mut 1–3) clones were seeded on day 0 in Geltrex-coated 96 well plates and then cultured for up to 4 days. Day 1 to 4 cells were collected by trypsinization and counted by flow cytometry. Cell numbers were expressed as ratios to the values obtained at day 1.

### Cell viability

WT 1 to 5 and Mut 1 to 3 clone cells were treated with 1.5 mM palmitic acid (solubilized in 2% fatty acid free BSA) + 22.2 mM glucose for 18 h. The cells were collected by trypsin, followed by labeling with Annexin V and propidium iodide for 10 min according to the manufacturer’s instructions (Molecular Probes Dead Cell Apoptosis Kits with Annexin V for Flow Cytometry, Invitrogen). Raw data were derived from a BD Accuri C6 plus flow cytometer with 488 nm laser excitation and FL-3 detection for PI and FL-1 detection for Annexin V-FITC staining. PI-positive cells (including both Annexin V-positive and negative cells) were considered necrotic. Annexin V-positive cells that were not PI-positive cells were counted as apoptotic cells. The percentage of total cell death was calculated as the sum of both apoptosis and necrosis.

### Mitochondrial respiration and extracellular acidification rates

Oxygen consumption rates (OCR) and extracellular acidification rates (ECAR) from WT 1 to 5 and Mut 1 to 3 cell clones were measured using the Extracellular Flux Analyzer XFe96 (Seahorse Bioscience) as previously described (44). WT 1 to 5 and Mut 1 to 3 cells (5 × 10^4^ per well, six replicates for each condition) were seeded directly in a Geltrex ECM Matrix-coated 96-well Seahorse plate. After culturing for 3 days, the clonal cells were exposed to assay medium containing 5.5 mM glucose (Seahorse Bioscience) (pH adjusted to 7.4) for 60 min at 37 °C before the microplate was inserted into the device. Then 2 μM each of oligomycin, FCCP, and the combination of rotenone and antimycin A were sequentially added into the medium at certain time points as shown, to determine ATP-coupled OCR, maximal OCR, and protein leak of the WT and Mut cells, respectively. All OCR measurements were corrected for non-mitochondrial OCR, and ECAR was measured in parallel.

### Mitochondrial membrane potential

WT 1 to 3 and Mut 1 to 3 clone cells were incubated with or without 1.5 mM palmitic acid + 22.2 mM glucose for 18 h and then stained with 4 μM JC-1 (Sigma-Aldrich) for 20 min in BSA-free culture medium. Cells were washed with PBS, followed by trypsinization for 5 min and analysis with flow cytometry. FL2/FL1 signal ratios were calculated as a measure of the inner mitochondrial membrane potential and then expressed as ratios to the WT control group.

### Mitochondrial superoxide production

Mitochondrial superoxide production was determined using the MitoSOX Red probe (ThermoFisher). WT 1 to 3 and Mut 1 to 3 clone cells were exposed to 1.5 mM palmitic acid + 22.2 mM glucose for 18h and then incubated with 5 μM MitoSOX Red for 30 min prior to analysis. After trypsinization, the FL-2 fluorescence intensity was quantified by flow cytometry. Results were expressed as ratios to the WT control group.

### Insulin and proinsulin release and contents

WT 1 to 3 and Mut 1 to 3 clone cells, cultured in 48-well plates, were pre-incubated for 30 min in 300 μl Krebs-Ringer bicarbonate HEPES (KRBH) buffer containing 1.7 mM glucose supplemented with 0.1% BSA. The cells were subsequently incubated in low (1.7 mM) or high (17 mM) glucose KRBH buffer with 0.1% BSA for 30 min. The supernatant of each group was collected for insulin/proinsulin secretion analysis using the human insulin and proinsulin ELISA kits from Mercodia. For cell contents of insulin and proinsulin, the cells were sonicated in a 0.5% Triton X-100 buffer and contents were analyzed with the same ELISA kits. Results were normalized to total cell protein contents, which were obtained using the DC Protein Assay Kit from Bio-Rad.

### Immunofluorescence staining

Cells were cultured in an 8-well chamber slide for 48h. The cells were washed with PBS thrice and fixed with 4% (v/v) paraformaldehyde for 5 min at room temperature. Then the cells were permeabilized with 0.2% (v/v) Triton X-100 followed by blocking with 2% (w/v) BSA in PBS. The primary antibodies were diluted 1:300 in PBS containing 1% (w/v) BSA and exposed to the cells overnight at 4 °C. Cells were washed with PBS thrice and then incubated with sets of combined panels of Alexa Fluor-labeled secondary antibodies. DAPI was used for visualization of nuclei. After mounting, slides were observed by a laser scanning confocal microscope (Zeiss LSM 780). Primary antibodies are listed below: Guinea pig anti-insulin (1:1000, Fitzgerald), Rabbit anti-TIA1 (1:300, Cell Signaling Technology), mouse anti-G3BP1 (1:300, Santa Cruz), mouse anti-proinsulin (1:300, Santa Cruz), rabbit anti-chromogranin A (N-terminal) (1:300, Fitzgerald).

### Whole transcriptome analysis

Total RNA from WT 1 to 5 and Mut 1 to 3 clones was isolated using the Qiagen RNEasy Plus RNA isolation kit. The RNA was then Illumina-sequenced, and gene ontology analysis was performed as previously described (44). RNA sequencing and bioinformatic analysis were performed by Novogene Sequencing.

### Quantitative real-time PCR analysis

Total RNA was collected from WT 1 to 5 and Mut 1 to 3 clone cells using the Qiagen RNeasy kit (Qiagen). The iScript Reverse Transcription Supermix Kit (Bio-Rad) was used to generate cDNA from the extracted RNA. Real-time PCR was performed using the Roche Light Cycler System and the FastStart DNA Master DNA SYBR Green I kit (Roche Diagnostics). Values were normalized to the relative amounts of GAPDH. Primer sequences are listed in Table S5.

### Glucagon contents

For cell contents of glucagon, the cells incubated in low glucose for 30 min were sonicated in a 0.5% Triton X-100 buffer and contents were analyzed with the glucagon ELISA kit from Mercodia. Results were normalized to total cell protein contents, which were collected using the DC Protein Assay Kit from Bio-Rad.

### Sub-plasma membrane Ca^2+^ imaging and analysis of total cell cAMP levels

Measurements of the cytoplasmic Ca^2+^ concentration beneath the plasma membrane were made with Fluo-5F in a total internal reflection fluorescence (TIRF) setup consisting of an Eclipse Ti microscope with a 60 × 1.45 oil-immersion NA-objective (Nikon), using 491 nm laser excitation (Cobolt AB) and fluorescence detection at 530 nm (35 nm half band-width) with an Orca-ER CCD camera (Hamamatsu). Total cell cAMP contents were analyzed using a cAMP Enzyme Immunoassay Kit according to the instructions of the manufacturer (Sigma Aldrich).

### RNA immunoprecipitation

WT 1 to 3 and Mut 1 to 3 cells were untreated or incubated for 6 h with 1.5 mM palmitate and 22 mM glucose. The cells (10^7^) were then RNA immunoprecipitated using the RiboCluster Profiler RIP-Assay Kit (Medical & Biological Laboratories Co), the rabbit anti-human TIA1 antibody (ab140595, Abcam), an antibody previously verified to specifically immunoprecipitate TIA1 (48), and Protein A Sepharose CL-4B beads (Cytiva) according to the instructions of the manufacturer. Following RNA isolation real-time RT-PCR was performed using the iTaq Universal SYBR Green One-Step Kit (Bio-Rad). Sequences of PCR primers are shown in Table S5. The Ct value of pre-immune rabbit IgG was subtracted from the corresponding anti-TIA1 Ct value. Results were then normalized to corresponding control.

### Lentivirus anti-*MYC* shRNA transduction of EndoC-βH1 cells

Lentiviral particles expressing GFP under the CMV promoter (pLenti-mGFP-Puro) were obtained from OriGene Technologies, Inc, with a scramble or an shRNA sequence targeting human *MYC* (CAT#: RC201611L4V). A mixture of WT 1 to 3 and Mut 1 to 3 clonal cells was transduced with the aforementioned shRNA lentiviral particles at a multiplicity of infection (MOI) of 50 for 36 h. Subsequently, the cells were replaced with fresh culture medium for an additional 48 h prior to qPCR analysis. The changes in fluorescence were observed using a fluorescence microscope throughout this time period.

### Alpha1-antitrypsin and liraglutide treatment

Alpha1-antitrypsin (A6150, Merck) is encoded by the SerpinA1 gene. 0.5 mg/ml Alpha1-Antitrypsin was used to treat WT 1 to 3 and Mut 1 to 3 clone cells for 4 days 100 nM liraglutide (AdipoGen Life Science) was used to treat WT 1 to 3 and Mut 1 to 3 clone cells for 14 days before further experiments.

### Statistics

Results are presented as means ± SEM and were depicted and analyzed using the Prism 10 software (Graphpad.com). Normal Gaussian distribution was assumed and equal deviations were verified using the Brown-Forsythe test. In experiments with multiple comparisons results, were analyzed using one-way ANOVA (repeated measurements) followed by Šídák's multiple comparisons *post hoc* test. In cases where only two groups were compared, Student’s two-sided *t* test was used.

## Data availability

The RNAseq dataset entitled "RNA-seq of human beta-cell line EndoC-betaH1 with and without single nucleotide replacement in the human TIA1 gene (E384 K mutation)” has been uploaded to EMBL-EBI ArrayExpress (accession number E-MTAB-16316).

## Supporting information

This article contains supporting information.

## Conflict of interest

The authors declare the following financial interests/personal relationships, which may be considered as potential competing interests: Å. S. has received lecture and consultancy fees from Novo-Nordisk, the manufacturer of liraglutide. No other potential conflicts of interest relevant to this article were reported.
