# Supplementary material for: The Welander TIA1 mutation dedifferentiates insulin-producing cells: Reversal by a GLP-1 receptor agonist[image]
Source: J Biol Chem. 2026 Mar 3;302(4):111336. doi: 10.1016/j.jbc.2026.111336 (PMC13054421; doi:10.1016/j.jbc.2026.111336)
Supplement: Supplementary Material — 1 [file mmc1.pdf]

Suppl. Figure 1

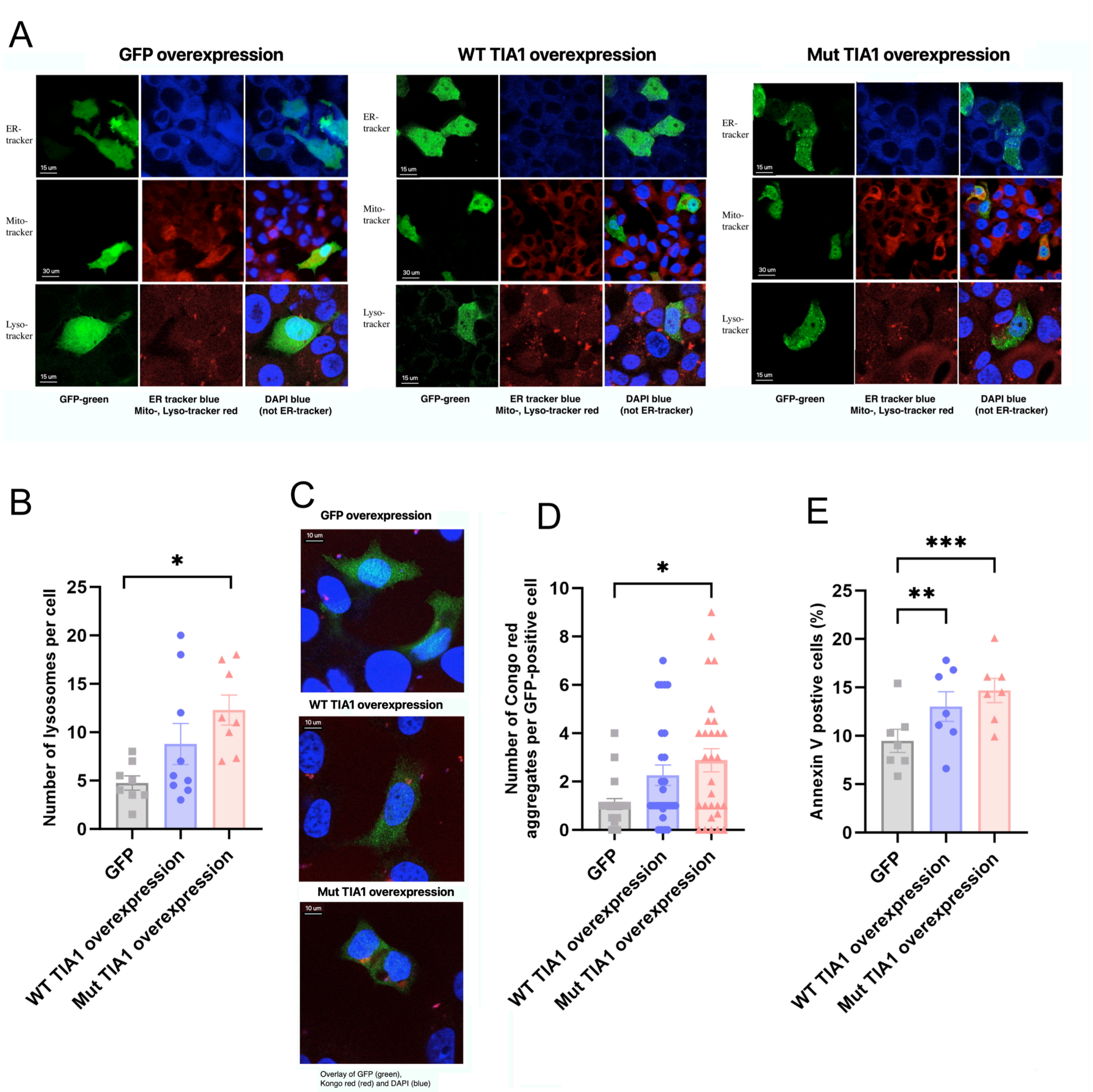

Suppl. Fig. 1

Subcellular localization, aggregate formation, and apoptosis of WT and mutant TIA1 transient overexpression in EndoC-βH1 cells. **(A)** GFP-tagged WT or Mut TIA1 constructs were transiently overexpressed in EndoC-βH1 cells. Eighteen hours after lipofection, GFP fluorescence for GFP vector, WT TIA1 or Mut TIA1, and its co-localization with ER, mitochondrial or lysosomal markers, were analyzed using confocal microscopy. **(B)** The number of lysosomes per cell was quantified in GFP, WT TIA1 and Mut TIA1 overexpressing EndoC-βH1 cells. 8-9 cells were analyzed in independent experiments and results are means ± SEM. * denotes p<0.01 using one-way ANOVA and the Šídák *post-hoc* test. **(C)** GFP vector, WT TIA1 and Mut TIA1 overexpressing EndoC-βH1 cells were stained with Congo red and DAPI and then observed under the confocal microscope. **(D)** Quantification of Congo red aggregates per cell (mean ± SEM) was made. 19-30 cells were analyzed in independent experiments and results are means ± SEM. * denotes p<0.01 using one-way ANOVA and the

Šídák *post-hoc* test. **(E)** Annexin V based apoptosis rates were assessed 18 hours after transfection in GFP, WT and Mut TIA1-overexpressing cells by flow cytometry. Result was analyzed in 7 independent experiments ** and **** denote $p<0.01$ and 0.0001, respectively, using one-way ANOVA and the Šídák *post-hoc* test.

Suppl. Figure 2

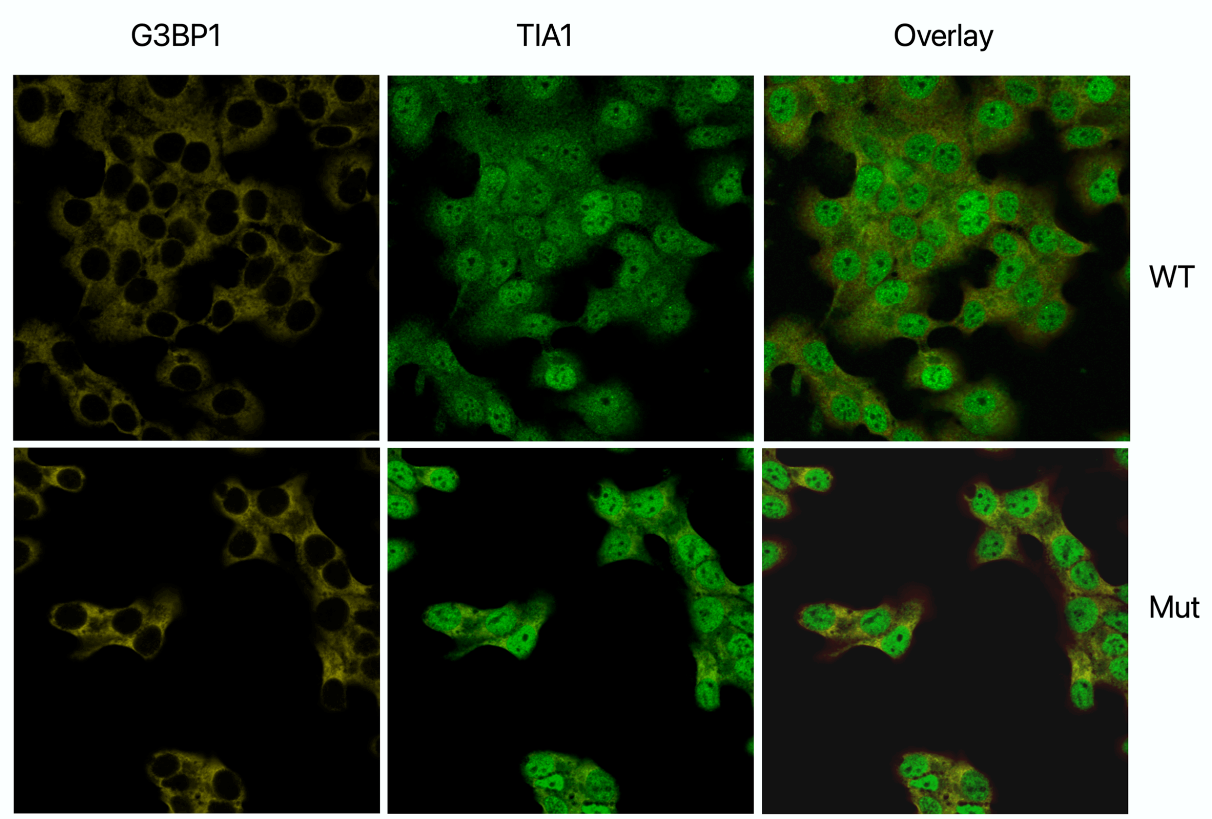

Suppl. Figure 2.
Subcellular localization of TIA1 and G3BP1 in WT 1 and Mut 1 cell clones. WT 1 and Mut 1 clonal cells were kept at basal conditions and then analyzed by confocal microscopy for G3BP1 and TIA1 subcellular staining patterns.
