# Supplementary material for: The Welander TIA1 mutation dedifferentiates insulin-producing cells: Reversal by a GLP-1 receptor agonist[image]
Source: J Biol Chem. 2026 Mar 3;302(4):111336. doi: 10.1016/j.jbc.2026.111336 (PMC13054421; doi:10.1016/j.jbc.2026.111336)
Supplement: Supplementary Material 2 [file mmc2.docx]

***Plasmid construction for transient overexpression***

Plasmids pFRT_DESTFLAGHA_eGFP (Addgene plasmid # 26362) (1) and pFRT_TO_eGFP_TIA1 (Addgene plasmid # 106094) (2) were obtained from Addgene (Watertown, MA, USA). Point mutation (E384K) in TIA1 coding sequence from pFRT_TO_eGFP_TIA1 plasmid was generated by site-directed mutagenesis using the Q5 Site-Directed Mutagenesis Kit (New England Biolabs, MA, USA) with the following primers: forward 5′- GGCAGGGTATaAAACCCAGTG -3′ and reverse 5′- ACTCGATACCCAGAAGGC-3′. Vectors were control sequenced using Eurofins custom DNA sequencing service. DNA sequences were analyzed using the SnapGene 5.2.5 software.

***Transient transfection***

For transfection of pFRT_DESTFLAGHA_eGFP (GFP-vector), pFRT_TO_eGFP_TIA1 (wild-type) and mutated pFRT_TO_eGFP_TIA1 (Mutated), 3x10^5^ EndoC-βH1 cells were resuspended in 100 µL Opti-MEM^TM^ I reduced serum medium (Thermo Fisher Scientific), containing 25 ng plasmid DNA and 0.5 µL Lipofectamine 2000 (Thermo Fisher Scientific), and seeded either onto the center of a 25 mm coverslip pre-coated with Geltrex Matrix (for imaging), or onto Geltrex-coated 48-well plates (for Annexin V staining). After 4 h cell culture medium was added and cells were cultured for another 16 h before assessment.

***Confocal microscopy of endoplasmic reticulum (ER), mitochondria, lysosomes and amyloid in GFP-expressing cells***

To study any putative co-localization between TIA1 and different cellular compartments/organelles, green fluorescent protein (GFP) subcellular localization was compared to that of ER, lysosomes, mitochondria and amyloid. For ER analysis, transiently transfected cells were vital stained using ER tracker Blue-White DPX (Molecular Probes) at 5 μM for 20 min. Nuclei were stained using bisbenzimide (10 μg/ml). Cells were then washed with PBS and analyzed by confocal microscopy using a Nikon TE2000 three laser C1-confocal microscope. For mitochondria, lysosomes and amyloid, cells were stained with Mitotracker red (5 μM, Molecular Probes), neutral red (5 μM, Sigma) and Congo red (5 μM), respectively. Cells were then fixed in 4% paraformaldehyde for 2 min and mounted using EverBrite Hardset Mounting Medium containing DAPI (Biotium). Confocal microscopy was performed as given above. The subcellular location of GFP as well as the fluorescent probes was studied using a 60x oil lens and 440, 530 and 650 nm detectors.

***Apoptosis rates***

Transiently transfected cells were harvested by mild trypsinization and then stained with Annexin V, CF 640R conjugate (Biotium) for 10 min according to the instructions of the manufacturer. This was followed by flow cytometry analysis using a BD Accuri C6 Plus instrument. Cells with increased FL-1 fluorescence were gated as GFP-positive cells and used for FL-4 analysis (Annexin V).

References

1. Spitzer. J.I., Ugras, S., Runge, S., Decarolis, P., Antonescu, C., Tuschl, T., et al. (2011) and protein levels of FUS, EWSR1, and TAF15 are upregulated in liposarcoma. *Genes Chromosomes Cancer* **50**, 338-47
2. Meyer, C., Garzia, A., Mazzola, M., Gerstberger, S., Molina, H., Tuschl, T. (2018) The TIA1 RNA-Binding Protein Family Regulates EIF2AK2-Mediated Stress Response and Cell Cycle Progression. *Mol. Cell* **69**, 622-635.e6
