# Supplementary material for: The Welander TIA1 mutation dedifferentiates insulin-producing cells: Reversal by a GLP-1 receptor agonist[image]
Source: J Biol Chem. 2026 Mar 3;302(4):111336. doi: 10.1016/j.jbc.2026.111336 (PMC13054421; doi:10.1016/j.jbc.2026.111336)
Supplement: Supplementary Material 3 [file mmc3.pptx]

## Slide 1
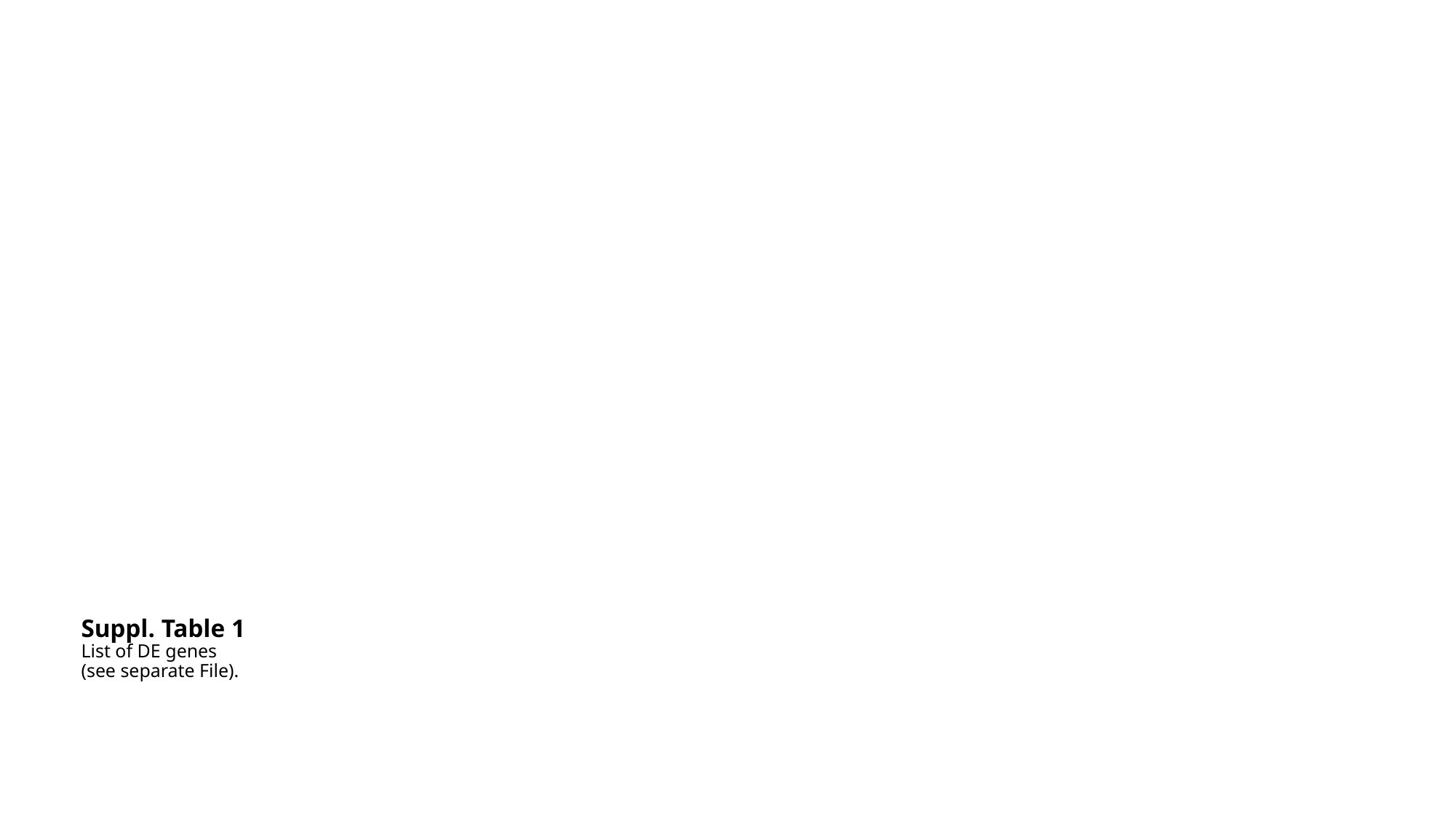

# Suppl. Table 1 List of DE genes (see separate File).

## Slide 2
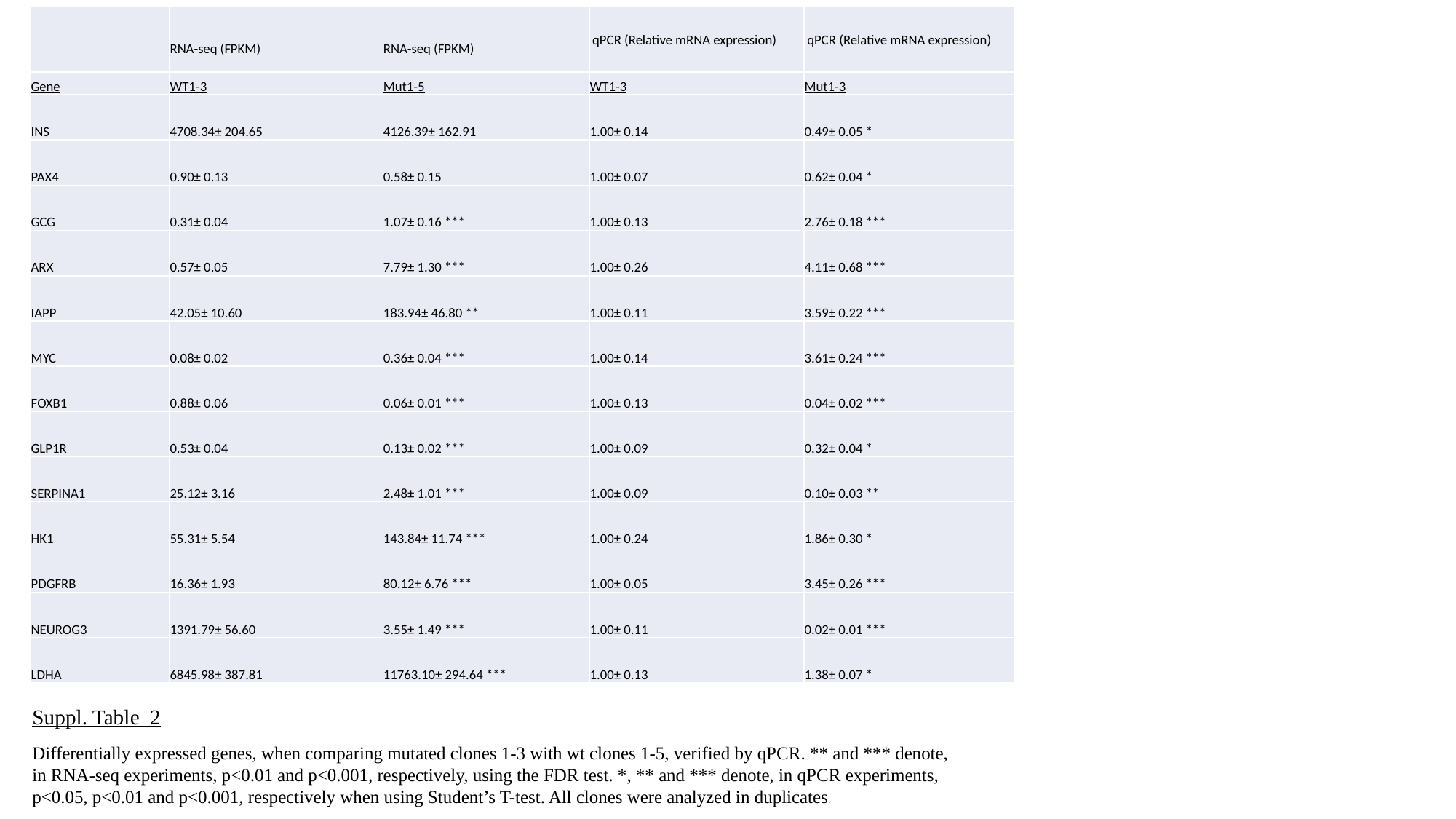

| | RNA-seq (FPKM) | RNA-seq (FPKM) | qPCR (Relative mRNA expression) | qPCR (Relative mRNA expression) |
| --- | --- | --- | --- | --- |
| Gene | WT1-3 | Mut1-5 | WT1-3 | Mut1-3 |
| INS | 4708.34± 204.65 | 4126.39± 162.91 | 1.00± 0.14 | 0.49± 0.05 \* |
| PAX4 | 0.90± 0.13 | 0.58± 0.15 | 1.00± 0.07 | 0.62± 0.04 \* |
| GCG | 0.31± 0.04 | 1.07± 0.16 \*\*\* | 1.00± 0.13 | 2.76± 0.18 \*\*\* |
| ARX | 0.57± 0.05 | 7.79± 1.30 \*\*\* | 1.00± 0.26 | 4.11± 0.68 \*\*\* |
| IAPP | 42.05± 10.60 | 183.94± 46.80 \*\* | 1.00± 0.11 | 3.59± 0.22 \*\*\* |
| MYC | 0.08± 0.02 | 0.36± 0.04 \*\*\* | 1.00± 0.14 | 3.61± 0.24 \*\*\* |
| FOXB1 | 0.88± 0.06 | 0.06± 0.01 \*\*\* | 1.00± 0.13 | 0.04± 0.02 \*\*\* |
| GLP1R | 0.53± 0.04 | 0.13± 0.02 \*\*\* | 1.00± 0.09 | 0.32± 0.04 \* |
| SERPINA1 | 25.12± 3.16 | 2.48± 1.01 \*\*\* | 1.00± 0.09 | 0.10± 0.03 \*\* |
| HK1 | 55.31± 5.54 | 143.84± 11.74 \*\*\* | 1.00± 0.24 | 1.86± 0.30 \* |
| PDGFRB | 16.36± 1.93 | 80.12± 6.76 \*\*\* | 1.00± 0.05 | 3.45± 0.26 \*\*\* |
| NEUROG3 | 1391.79± 56.60 | 3.55± 1.49 \*\*\* | 1.00± 0.11 | 0.02± 0.01 \*\*\* |
| LDHA | 6845.98± 387.81 | 11763.10± 294.64 \*\*\* | 1.00± 0.13 | 1.38± 0.07 \* |
Suppl. Table 2
Differentially expressed genes, when comparing mutated clones 1-3 with wt clones 1-5, verified by qPCR. ** and *** denote,
in RNA-seq experiments, p<0.01 and p<0.001, respectively, using the FDR test. *, ** and *** denote, in qPCR experiments,
p<0.05, p<0.01 and p<0.001, respectively when using Student’s T-test. All clones were analyzed in duplicates.

## Slide 3
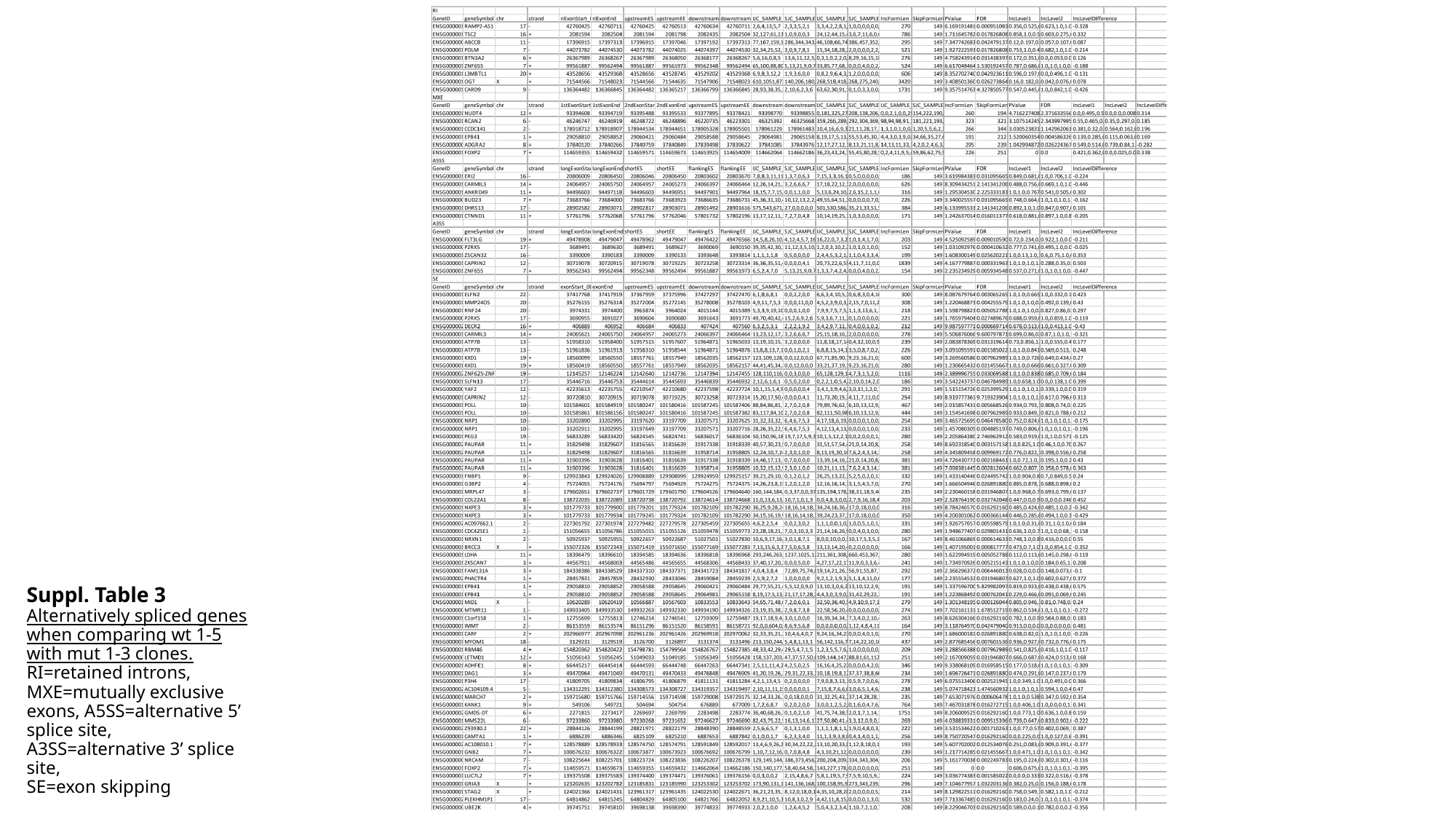

# Suppl. Table 3 Alternatively spliced genes when comparing wt 1-5 with mut 1-3 clones. RI=retained introns, MXE=mutually exclusive exons, A5SS=alternative 5’ splice site, A3SS=alternative 3’ splice site, SE=exon skipping

## Slide 4
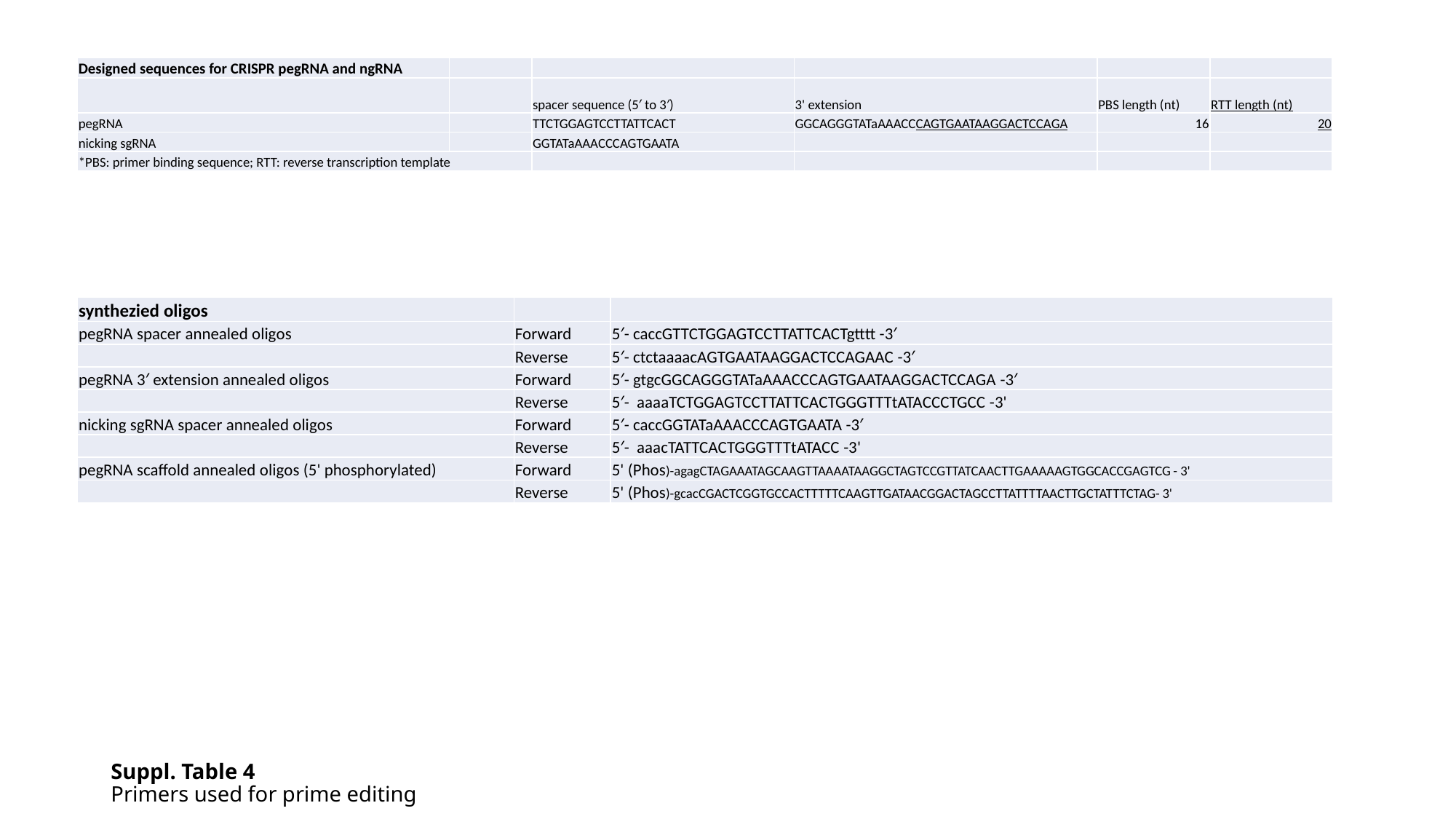

| Designed sequences for CRISPR pegRNA and ngRNA | | | | | |
| --- | --- | --- | --- | --- | --- |
| | | spacer sequence (5′ to 3′) | 3' extension | PBS length (nt) | RTT length (nt) |
| pegRNA | | TTCTGGAGTCCTTATTCACT | GGCAGGGTATaAAACCCAGTGAATAAGGACTCCAGA | 16 | 20 |
| nicking sgRNA | | GGTATaAAACCCAGTGAATA | | | |
| \*PBS: primer binding sequence; RTT: reverse transcription template | | | | | |
| synthezied oligos | | |
| --- | --- | --- |
| pegRNA spacer annealed oligos | Forward | 5′- caccGTTCTGGAGTCCTTATTCACTgtttt -3′ |
| | Reverse | 5′- ctctaaaacAGTGAATAAGGACTCCAGAAC -3′ |
| pegRNA 3′ extension annealed oligos | Forward | 5′- gtgcGGCAGGGTATaAAACCCAGTGAATAAGGACTCCAGA -3′ |
| | Reverse | 5′- aaaaTCTGGAGTCCTTATTCACTGGGTTTtATACCCTGCC -3' |
| nicking sgRNA spacer annealed oligos | Forward | 5′- caccGGTATaAAACCCAGTGAATA -3′ |
| | Reverse | 5′- aaacTATTCACTGGGTTTtATACC -3' |
| pegRNA scaffold annealed oligos (5' phosphorylated) | Forward | 5' (Phos)-agagCTAGAAATAGCAAGTTAAAATAAGGCTAGTCCGTTATCAACTTGAAAAAGTGGCACCGAGTCG - 3' |
| | Reverse | 5' (Phos)-gcacCGACTCGGTGCCACTTTTTCAAGTTGATAACGGACTAGCCTTATTTTAACTTGCTATTTCTAG- 3' |
# Suppl. Table 4 Primers used for prime editing

## Slide 5
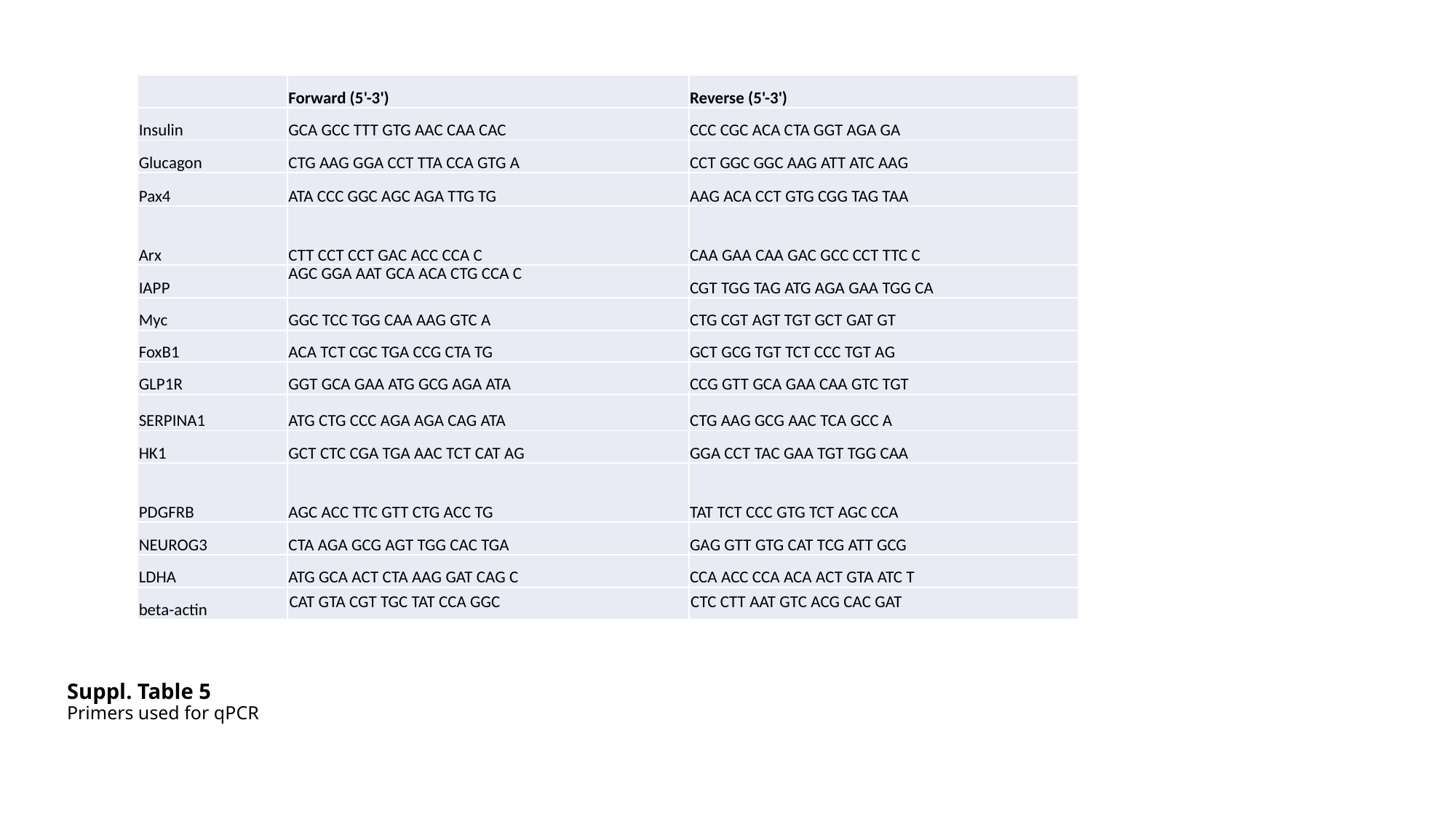

| | Forward (5'-3') | Reverse (5'-3') |
| --- | --- | --- |
| Insulin | GCA GCC TTT GTG AAC CAA CAC | CCC CGC ACA CTA GGT AGA GA |
| Glucagon | CTG AAG GGA CCT TTA CCA GTG A | CCT GGC GGC AAG ATT ATC AAG |
| Pax4 | ATA CCC GGC AGC AGA TTG TG | AAG ACA CCT GTG CGG TAG TAA |
| Arx | CTT CCT CCT GAC ACC CCA C | CAA GAA CAA GAC GCC CCT TTC C |
| IAPP | AGC GGA AAT GCA ACA CTG CCA C | CGT TGG TAG ATG AGA GAA TGG CA |
| Myc | GGC TCC TGG CAA AAG GTC A | CTG CGT AGT TGT GCT GAT GT |
| FoxB1 | ACA TCT CGC TGA CCG CTA TG | GCT GCG TGT TCT CCC TGT AG |
| GLP1R | GGT GCA GAA ATG GCG AGA ATA | CCG GTT GCA GAA CAA GTC TGT |
| SERPINA1 | ATG CTG CCC AGA AGA CAG ATA | CTG AAG GCG AAC TCA GCC A |
| HK1 | GCT CTC CGA TGA AAC TCT CAT AG | GGA CCT TAC GAA TGT TGG CAA |
| PDGFRB | AGC ACC TTC GTT CTG ACC TG | TAT TCT CCC GTG TCT AGC CCA |
| NEUROG3 | CTA AGA GCG AGT TGG CAC TGA | GAG GTT GTG CAT TCG ATT GCG |
| LDHA | ATG GCA ACT CTA AAG GAT CAG C | CCA ACC CCA ACA ACT GTA ATC T |
| beta-actin | CAT GTA CGT TGC TAT CCA GGC | CTC CTT AAT GTC ACG CAC GAT |
# Suppl. Table 5 Primers used for qPCR
